# Glutathione Synthesis *via* the Cystine/Glutamate Transporter Promotes the Formation of Tertiary Lymphoid Structures in the Kidney

**DOI:** 10.1681/ASN.0000000825

**Published:** 2025-08-08

**Authors:** Hiroyuki Arai, Yuki Sugiura, Shinya Yamamoto, Takahisa Yoshikawa, Yuta Matsuoka, Rae Maeda, Hiroyuki Neyama, Ryo Kamimatsuse, Shima Goto, Keisuke Taniguchi, Naoya Toriu, Makiko Kondo, Yuki Sato, Shingo Fukuma, Motoko Yanagita

**Affiliations:** 1 Department of Nephrology, Graduate School of Medicine, Kyoto University, Kyoto, Japan; 2 Center for Cancer Immunotherapy and Immunobiology, Graduate School of Medicine, Kyoto University, Kyoto, Japan; 3 Department of Drug Discovery Medicine, Graduate School of Medicine, Kyoto University, Kyoto, Japan; 4 Preclinical Research Unit, Drug Research Division, Sumitomo Pharma Co., Ltd, Osaka, Japan; 5 Institute for the Advanced Study of Human Biology (WPI-ASHBi), Kyoto University, Kyoto, Japan; 6 Department of Nephrology, Japanese Red Cross Otsu Hospital, Shiga, Japan; 7 Human Health Sciences, Graduate School of Medicine, Kyoto University, Kyoto, Japan

**Keywords:** AKI, chronic inflammation, lymphocytes, biomarkers, metabolomics

## Abstract

**Key Points:**

- Glutathione accumulated in tertiary lymphoid structures (TLS), where dendritic cells and fibroblasts specifically expressed cystine/glutamate transporter.
- Pharmacologic inhibition of the cystine/glutamate transporter prevented the formation of TLS.
- Urinary glutathione concentrations efficiently detected the presence of TLS in the kidney in mice and humans.

**Background:**

Tertiary lymphoid structure (TLS), an ectopic lymphoid tissue induced under chronic inflammation, develops in various kidney diseases and is associated with poor prognosis. The immune system requires metabolic resources to support immune function and lymphocyte proliferation. Hence, dramatic metabolic alterations presumably occur during the formation of TLS. However, it remains unclear whether metabolic remodeling occurs during this formation and its underlying mechanism.

**Methods:**

In a murine model of TLS in the kidney, we used imaging mass spectrometry and metabolome analysis to investigate the metabolic pathway that characterizes TLS. We also performed *in situ* hybridization with immunofluorescence and pharmacologic inhibition to explore the expression and function of the key molecules governing the pivotal metabolic pathway. We analyzed urine samples from mice and humans to explore the metabolites estimating the presence of TLS in the kidney.

**Results:**

Significant glutathione accumulation and depletion of cysteine, which is essential for glutathione synthesis, was observed specifically within TLS. The kidneys with TLS exhibited higher glutathione concentrations than healthy kidneys. TLS also showed significant accumulation of 4-hydroxynonenal and 8-hydroxy-2′ -deoxyguanosine, markers of oxidative stress. Dendritic cells and fibroblasts within TLS expressed the cystine/glutamate transporter, which regulates glutathione synthesis, and supplied synthesized glutathione to lymphocytes, which lacked its expression. Pharmacologic inhibition of the cystine/glutamate transporter prevented the formation of TLS in the kidney. Furthermore, enhanced glutathione synthesis within TLS was reflected in elevated urinary glutathione concentrations in both mice and humans, which effectively detected the presence of TLS in the kidney in IgA nephropathy patients.

**Conclusions:**

Glutathione significantly accumulated within TLS in the kidney. Inhibition of the cystine/glutamate transporter prevented the formation of TLS. Urinary glutathione served as a biomarker to detect TLS in the kidney.

## Visual Abstract

**Figure:**
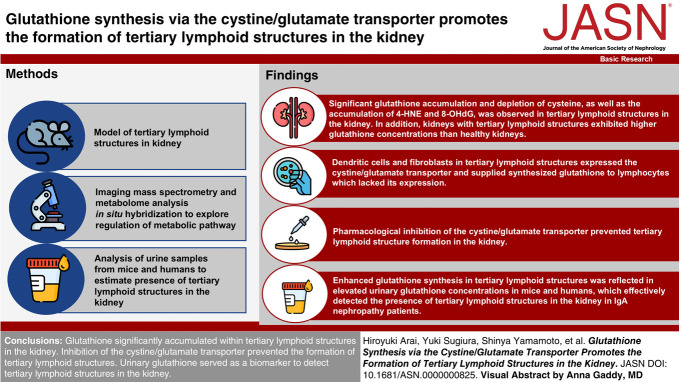

## Introduction

Tertiary lymphoid structures (TLS, also known as tertiary lymphoid tissues, tertiary lymphoid organs, or ectopic lymphoid tissues) are ectopic lymphoid structures that are induced in nonlymphoid organs by several stimuli, including autoimmunity, infection, and aging.^1^ TLS are mainly composed of lymphocytes and are supported, structurally and functionally, by fibroblasts with distinct phenotypes. TLS serve as local sites of adaptive immune responses in which vigorous lymphocyte proliferation and cytokine production occur. TLS are formed in aged kidneys as well as in various kidney diseases, such as IgA nephropathy,^2^ lupus nephritis,^3^ and kidney transplants.^4^ Recently, we reported that TLS amplify inflammation by providing a microenvironment that allows intense interactions between kidney parenchymal and immune cells, leading to failed repair of the kidneys.^5,6^

The immune system requires substantial metabolic resources for specific immune functions and lymphocyte proliferation. For example, glutathione is essential for the proliferation and activation of T cells,^7^ and interference of glutathione metabolism prevents normal immune responses.^8^ To meet the metabolic demand of immune cells, systemic metabolic remodeling is required.^9^ In the formation of TLS, because an enormous number of immune cells are ectopically recruited to nonlymphoid organs, it is speculated that dramatic metabolic remodeling occurs in immune cells in the organ. However, the mechanism of metabolic remodeling required for the formation of TLS remains unclear.

To elucidate this, we investigated the metabolic microenvironment within TLS in the kidney using imaging mass spectrometry and metabolome analysis. We hypothesized that specific metabolic pathways are significantly altered within TLS and play a crucial role in its formation.

## Methods

### Analysis of Human Specimens

All human specimens were analyzed after obtaining informed consent with the approval of the Ethics Committee at Kyoto University Hospital (Kyoto, Japan). Forty-six patients with IgA nephropathy at Kyoto University Hospital who underwent kidney biopsy between 2014 and 2018 were recruited (Table 1 and Supplemental Figure 1). Metabolome analysis of urine samples from the patients was conducted, as described in the Supplemental Materials.

**Table 1:** Background of IgA nephropathy patients with or without tertiary lymphoid structures in the kidney

Characteristics	Total (*n*=46)	Without Tertiary Lymphoid Structures (*n*=28)	With Tertiary Lymphoid Structures (*n*=18)
Age, yr	40 (26–49)	32 (24–42)	53 (35–70)
Sex, men/women	17/29	7/21	10/8
BMI, kg/m^2^	21.3 (18.9–22.9)	20.9 (18.5–22.4)	21.9 (20.0–23.7)
Serum creatinine, mg/dl	0.96 (0.61–1.09)	0.73 (0.58–0.92)	1.31 (0.84–1.83)
eGFR, ml/min per 1.73 m^2^	75 (53–94)	90 (76–102)	52 (29–64)
UPCR, g/gCr	0.98 (0.23–0.82)	0.45 (0.18–0.63)	1.82 (0.30–2.91)
Diabetes, *n* (%)	3 (7)	1 (4)	2 (11)
Hypertension, *n* (%)	12 (26)	1 (4)	11 (61)

Data are median (25th–75th percentile) or percentage. Categorical and continuous variables were analyzed by Pearson chi-square test and Wilcoxon rank-sum test, respectively. BMI, body mass index; UPCR, urinary protein-to-creatinine ratio.

**Figure 1:**
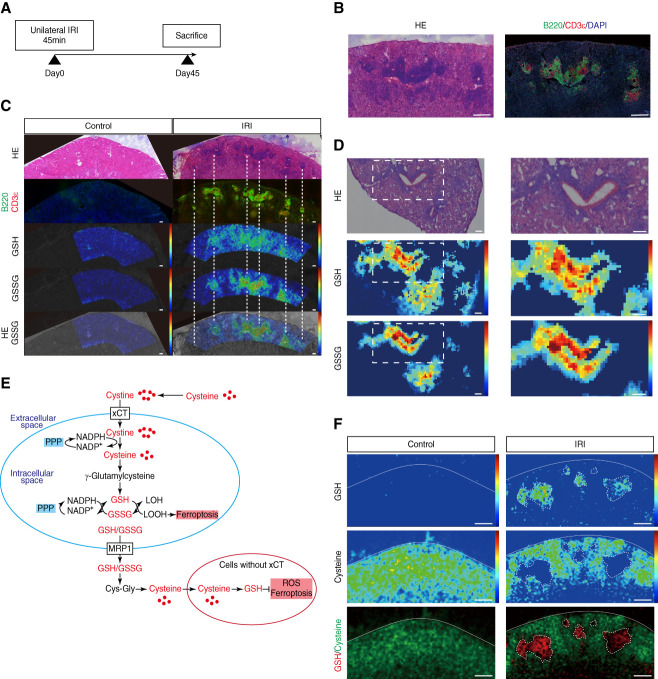

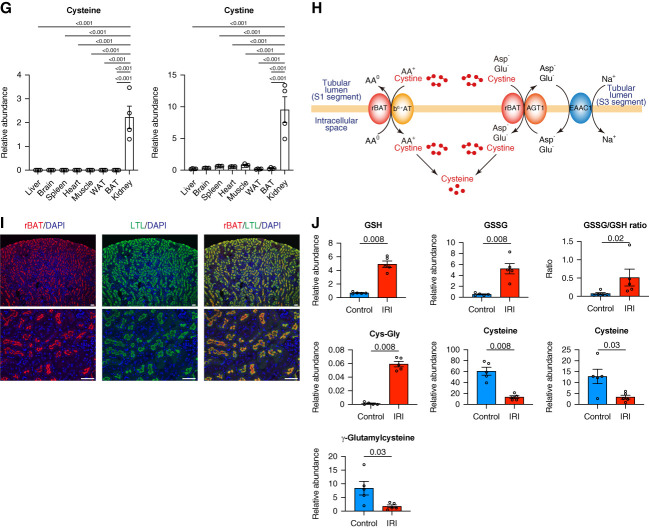
**TLS in the kidney consume cyst(e)ine to synthesize glutathione.** (A) Experimental protocol for (B–D, F, and J). (B) HE staining and immunofluorescence of B220 (green), CD3ε (red), and DAPI (blue) of the kidneys 45 days after IRI. (C) HE staining, immunofluorescence of B220 (green) and CD3ε (red), and MALDI-TOF imaging mass spectrometry of GSH (*m*/*z* 306.0) and GSSG (*m*/*z* 611.1) in the kidneys 45 days after sham surgery and IRI. Sections used for imaging mass spectrometry were stained by HE after imaging mass spectrometry procedure, and their serial sections were analyzed by immunofluorescence. (D) Tandem (MS/MS) imaging mass spectrometry of GSH and GSSG in the kidneys 45 days after IRI. Data were processed by the SCiLS Lab software (Bruker Daltonics, Billerica, MA). The color bar indicates peak intensity levels at m/z 306.0 (GSH) or 611.1 (GSSG), with red and blue representing high and low signals, respectively. The regions delineated by dotted squares were enlarged for detailed demonstration on the right. (E) Scheme of intercellular glutathione metabolism. (F) Orbitrap imaging mass spectrometry of cysteine and GSH in the kidneys 45 days after sham surgery and IRI. Kidney cortex and TLS are encircled by solid and dotted lines, respectively. (G) Metabolome analysis of cysteine and cystine in the liver, brain, spleen, heart, skeletal muscle (muscle), WAT, BAT, and kidney (*n*=4, each). (H) Scheme of cystine transport mechanism on kidney tubules. (I) Immunofluorescence of rBAT (red), LTL (green), and DAPI (blue) in the healthy kidney. (J) Metabolome analysis of metabolites related to glutathione synthesis pathway and GSSG/GSH ratio in the kidneys 45 days after sham surgery and IRI (*n*=5 in each group). Values are means±SEM. Data were analyzed by (G) nonparametric multiple comparisons with Dunnett-type contrasts using the nparcomp R package with kidney as control and (J) Mann–Whitney *U* test. Scale bars: (B) 500 *µ*m, (C) 500 *µ*m, (D) 200 *µ*m, (F) 500 *µ*m, and (I) 50 *µ*m. BAT, brown adipose tissue; Cys-Gly, cysteine-glycine; DAPI, 4′,6-diamidino-2-phenylindole; GSH, glutathione; GSSG, glutathione disulfide; HE, hematoxylin-eosin; IRI, ischemia-reperfusion injury; LOH, lipid hydroxide (reduced form of lipid hydroperoxide); LOOH, lipid hydroperoxide; LTL, lotus tetragonobus lectin; MALDI-TOF, matrix-assisted laser desorption/ionization-time of flight; MS/MS, mass spectrometry; PPP, pentose phosphate pathway; rBAT, related to b0,+ amino acid transporter; ROS, reactive oxygen species; TLS, tertiary lymphoid structures; WAT, white adipose tissue.

### Imaging Mass Spectrometry

Matrix-assisted laser desorption/ionization time of flight imaging mass spectrometry (matrix-assisted laser desorption/ionization–imaging mass spectrometry) was performed on murine kidneys, as previously described.^10^ Image reconstruction was performed using FlexImaging 4.1 and Image Quest 1.1 software. Full details are provided in the Supplemental Methods; Supplemental Table 2.

### Study Approval

All animal experiments were approved by the Animal Research Committee, Graduate School of Medicine, Kyoto University (MedKyoto20187), and were conducted in accordance with the *Guide for the Care and Use of Laboratory Animals* (National Institutes of Health, Bethesda, MD). The sample sizes were determined based on preliminary experiments and previous studies using similar models. The chosen number of animals represents the minimum required to achieve reliable results while adhering to ethical standards and minimizing animal use in accordance with the Animal Research: Reporting of *In Vivo* Experiments checklists. All human specimens were procured and analyzed after obtaining written informed consent with the approval of the Ethics Committees of Graduate School of Medicine, Kyoto University (G562).

## Results

### Tertiary Lymphoid Structures Actively Synthesized Glutathione by Consuming Cystine and Cysteine

TLS were formed in aged injured kidneys after ischemia-reperfusion injury (IRI)^11^ and were detected as aggregates of B and T cells that were supported by fibroblasts with distinct phenotype (Figure 1, A and B). To visualize small-molecule metabolites localized within TLS, we used matrix-assisted laser desorption/ionization time of flight imaging mass spectrometry and found significant accumulation of glutathione (reduced form: glutathione [GSH], oxidized form: glutathione disulfide [GSSG]) within TLS (Figure 1C). Tandem mass spectrometry imaging detected the strong glutathione signals from the site of lymphocyte accumulation to adjacent sites (Figure 1D). GSH accumulation within TLS was also confirmed by desorption electrospray ionization–mass spectrometry imaging (Supplemental Figure 2). Signal of cysteine, which is required for glutathione synthesis (Figure 1E), was selectively depleted within TLS in injured kidneys, whereas it was uniformly distributed in healthy kidneys (Figure 1F).

**Figure 2:**
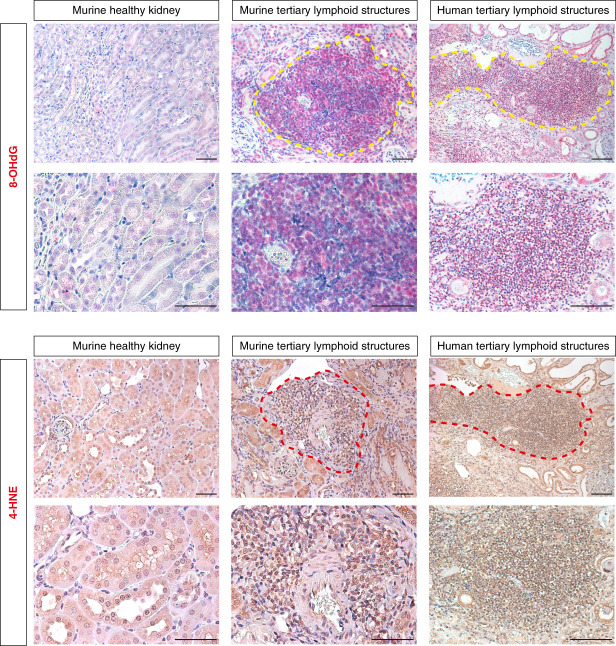
**8-OHdG and 4-HNE accumulate within TLS in the kidney of both mice and humans.** Immunohistochemistry of 8-OHdG and 4-HNE in murine and human kidneys with TLS and control kidneys. Human nephrectomized kidney samples in which TLS were confirmed by immunostaining were used. 8-OHdG and 4-HNE were detected by alkaline phosphatase (red) and DAB (brown) staining, respectively. Sections were counterstained with eosin. TLS are encircled by dotted lines. We investigated nephrectomized kidney samples from patients with renal cell carcinoma, focusing on the noncancerous areas of the kidney where TLS were observed. Scale bars: 50 *µ*m. 4-HNE, 4-hydroxynonenal; 8-OHdG, 8-hydroxy-2′-deoxyguanosine; DAB, 3,3′-diaminobenzidine.

Quantitative metabolome analysis of various healthy tissues revealed that the kidneys contained much more cysteine and cystine (oxidized from cysteine) than the other organs (Figure 1G). In the kidney, cystine is reabsorbed by transporters consisting of heterodimers of related to b0,+ amino acid transporter and either b0AT or AGT1 (Figure 1H).^12,13^ Indeed, related to b0,+ amino acid transporter expression was detected exclusively in the proximal tubules, indicating active cystine reabsorption under physiologic conditions (Figure 1I). These results suggest that the kidney is a cyst(e)ine-rich organ and that lymphocytes in TLS may actively consume cyst(e)ine to synthesize glutathione.

To comprehensively compare metabolite levels in the glutathione synthesis pathway (Figure 1E), we performed metabolome analysis of the kidney tissue. In accordance with the imaging mass spectrometry results, GSH and GSSG levels were significantly elevated in kidneys with TLS, accompanied by an increased GSSG/GSH ratio (Figure 1J). Cysteine-glycine, a breakdown product of extracellular glutathione (Figure 1E), was also elevated in kidneys with TLS, suggesting extracellular glutathione transport.^14^ By contrast, cystine, cysteine, and *γ*-glutamylcysteine, intermediate metabolites of the glutathione synthesis pathway (Figure 1E), were decreased in the kidneys with TLS. In addition, NADPH was decreased in the kidneys with TLS, which is consumed in the reduction of cystine to cysteine and GSSG to GSH. NADPH is produced through the pentose phosphate pathway (PPP) (Figure 1E, Supplemental Figure 3A). PPP intermediate products such as 6-phosphogluconate, ribulose-5P, sedoheptulose-7P, and glyceraldehide-3P were increased, and glucose-6P was decreased (Supplemental Figure 3B). From these findings and the fact that glutathione is essential for lymphocyte proliferation and immune responses,^7,8^ we hypothesized that abundant cystine/cysteine in the kidneys forms a unique metabolic microenvironment that supports ectopic lymphocyte proliferation, leading to the formation of TLS.

### Tertiary Lymphoid Structures Represented a Unique Microenvironment with High Oxidative Stress

We further investigated the redox status of TLS by immunostaining for two established markers of oxidative stress: 8-hydroxy-2′-deoxyguanosine (8-OHdG) and 4-hydroxynonenal (4-HNE). 8-OHdG and 4-HNE highly accumulated in both murine and human TLS in the kidney (Figure 2). To emphasize the unique redox microenvironment of TLS, we analyzed oxidative stress levels in immune cell subpopulations from TLS, spleen, and peripheral blood using the CellROX oxidative stress detection reagent. The mean fluorescence intensity of CellROX was significantly higher in immune cells from TLS than those from the spleen or peripheral blood (Supplemental Figure 4). Although we could not exclude the influence of different isolation procedures for each organ, along with the glutathione accumulation within TLS, these findings suggest that TLS in the kidney represent a unique microenvironment characterized by a dynamic equilibrium between oxidative stress and glutathione accumulation, which may play a pivotal role in regulating the formation of TLS and its function.

### Dendritic Cells and Fibroblasts within TLS Exclusively Expressed the Cystine/Glutamate Transporter, a Key Molecule in Glutathione Synthesis Pathway

System x_c_^−^ is the cystine/glutamate transporter that transports extracellular cystine into cells, and its transport activity is regulated by the light chain subunit encoded by *Slc7a11*.^15^ The rate-limiting step of glutathione synthesis is cystine import through the cystine/glutamate transporter (Figure 1E), and its expression is induced under oxidative stress and during cell proliferation.^15,16^ Because TLS are accompanied by high oxidative stress (Figure 2 and Supplemental Figure 4) and active cell proliferation,^11^ we analyzed the expression of the cystine/glutamate transporter within TLS.

**Figure 3:**
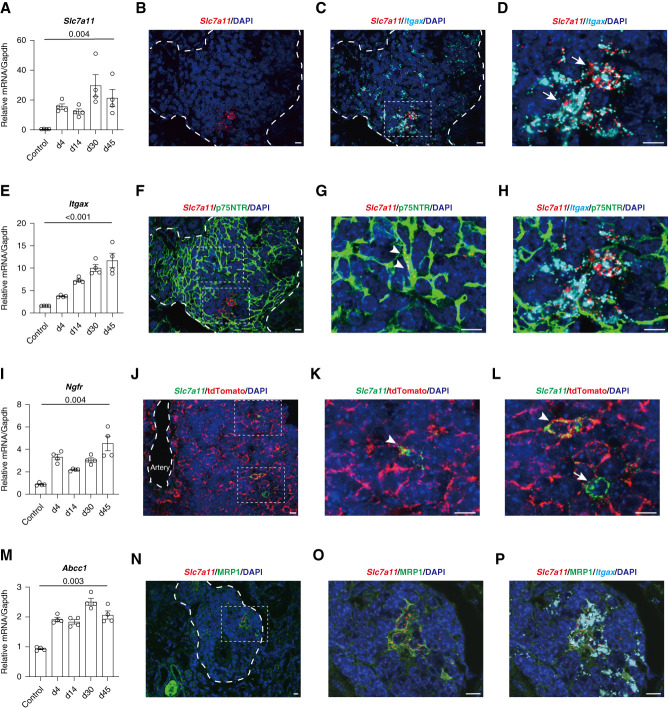

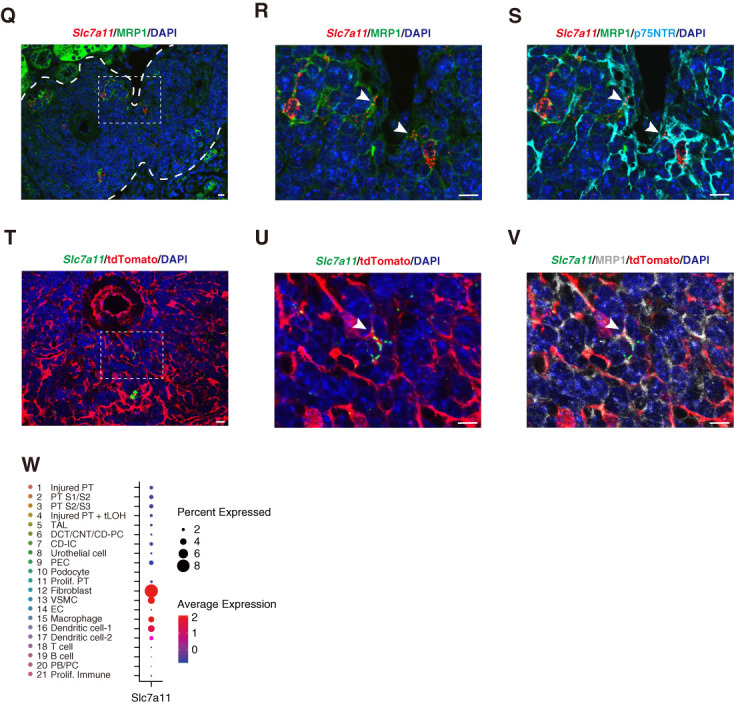
**Selective expression of *Slc7a11* and MRP1 in dendritic cells and fibroblasts within TLS in the kidney.** (A) *Slc7a11* mRNA levels in the kidneys at days 4, 14, 30, and 45 after sham surgery and IRI (*n*=4 at each timepoint). (B–D) ISH with RNAscope of *Slc7a11* (red) and *Itgax* (light blue) combined with DAPI staining (blue) in the kidneys 45 days after IRI. The region delineated by dotted squares in (C) is enlarged for detailed demonstration in (D). White arrows indicate *Slc7a11*^+^*Itgax*^+^cells. (E) *Itgax* mRNA levels in the kidneys at days 4, 14, 30, and 45 after sham surgery and IRI (*n*=4 at each timepoint). (F–H) Combination of ISH (*Slc7a11* [red] and *Itgax* [light blue]) and immunofluorescence (p75NTR [green] and DAPI [blue]) in the kidneys 45 days after IRI. The regions delineated by dotted squares in (F) are enlarged for detailed demonstration in (G) and (H). White arrowheads represent *Slc7a11*^+^p75NTR^+^cells. (I) *Ngfr* (p75NTR) mRNA levels in the kidneys at days 4, 14, 30, and 45 after sham surgery and IRI (*n*=4 at each timepoint). (J–L) ISH of *Slc7a11* (green) combined with DAPI staining (blue) in P0-Cre/tdTomato mice 45 days after IRI (red: tdTomato signals in P0-lineage cells). The regions delineated by dotted squares in (J) are enlarged for detailed demonstration in (K) and (L). Artery is encircled by dotted line. White arrows and arrowheads indicate *Slc7a11*^+^tdTomato^−^ cells and *Slc7a11*^+^tdTomato^+^cells, respectively. (M) *Abcc1* (MRP1) mRNA levels in the kidneys at days 4, 14, 30, and 45 after sham surgery and IRI (*n*=4 at each timepoint). (N–P) Combination of ISH (*Slc7a11* [red] and *Itgax* [light blue]) and immunofluorescence (MRP1 [green] and DAPI [blue]) in the kidneys 45 days after IRI. The region delineated by dotted square in (N) is enlarged for detailed demonstration in (O) and (P). (Q–S) A combination of ISH (*Slc7a11* [red]) and immunofluorescence (MRP1 [green], p75NTR [light blue], and DAPI [blue]) in the kidneys 45 days after IRI. The region delineated by dotted square in (Q) is enlarged for detailed demonstration in (R) and (S). White arrowheads represent *Slc7a11*^+^MRP1^+^p75ntr^+^cells. (T–V) A combination of ISH (*Slc7a11* [green]) and immunofluorescence (MRP1 [gray]) in P0-Cre/tdTomato mice 45 days after IRI (red: tdTomato signals in P0-lineage–labeled cells). The regions delineated by dotted squares in (T) are enlarged for detailed demonstration in (U) and (V). White arrowheads indicate *Slc7a11*^+^MRP1^+^tdTomato^+^cells. (W) Dot plots displaying expression patterns of Slc7a11 for each cluster by sn-RNAseq on kidneys 30 days after 45-minute IRI.^5^ (B, C, F, N, and Q) The areas encircled by the dashed line indicate TLS. Values are means±SEM. Data in (A, E, I, and M) were analyzed by nonparametric trend test. Scale bars (B–D, F–H, J–L, and N–V): 10 *µ*m. CD-IC, collecting duct, intercalated cell; CD-PC, collecting duct-principal cell; CNT, connecting tubule; EC, endothelial cell; ISH, *in situ* hybridization; p75NTR, p75 neurotrophin receptor; PB/PC, plasmablast/plasma cell; PC, phosphatidylcholine; PEC, parietal epithelial cell; PT, proximal tubule; TAL, thick ascending limb; tLOH, thin limbs of the loop of Henle; VSMC, vascular smooth muscle cell.

The expression of *Slc7a11* in the kidneys increased after IRI, reaching a peak at days 30 and 45, when mature TLS are formed (Figure 3A).^17^ The localization of the cystine/glutamate transporter could not be confirmed by commercially available antibodies because of their low sensitivity and specificity.^18,19^ Therefore, we used an RNAscope probe against *Slc7a11* and revealed its expression within TLS (Figure 3B). Interestingly, some *Slc7a11* signals colocalized with the *Itgax* RNAscope signal, a dendritic cell marker whose expression increased after IRI (Figure 3, C–E). Similarly, some *Slc7a11* RNAscope signals colocalized with p75 neurotrophin receptor, a marker of unique fibroblasts within TLS,^11^ whose expression (gene name: *Ngfr*) also increased after IRI (Figure 3, F–I). Analysis using *P0-Cre/R26 tdTomato* mice, which lineage labels resident fibroblasts^20^ as well as fibroblasts within TLS,^11^ also showed that some *Slc7a11* signals colocalized with tdTomato-positive fibroblasts within TLS (Figure 3, J–L, arrowheads). Based on RNAscope analysis, approximately 24% (mean) of dendritic cells and 14% (mean) of fibroblasts expressed the cystine/glutamate transporter (Supplemental Figure 5).

Contrary to our initial expectations, the cystine/glutamate transporter was expressed in very limited cell populations within TLS; intercellular glutathione transport from these cells to lymphocytes was expected.^14,21–24^ Expression of *Abcc1*, which encodes MRP1 that is responsible for extracellular glutathione efflux (Figure 1E),^25^ also increased after IRI (Figure 3M). MRP1 expression was shown to be localized to *Slc7a11*-positive dendritic cells (Figure 3, N–P) and fibroblasts within TLS (Figure 3, Q–S). Analysis on P0-Cre/R26 tdTomato mice also showed that tdTomato-positive fibroblasts within TLS coexpressed *Slc7a11* and MRP1 (Figure 3, T–V, arrowheads). In snRNAseq dataset of mouse kidneys with TLS,^5^ *Slc7a11* was strongly expressed in dendritic cells and fibroblasts (Figure 3W), consistent with RNAscope findings. Taken together, these findings suggest that glutathione synthesis within TLS may be distinctly regulated, with dendritic cells and fibroblasts that express the cystine/glutamate transporter playing an important role.

### Pharmacologic Inhibition of the Cystine/Glutamate Transporter Suppressed the Formation of Tertiary Lymphoid Structures

Glutathione is essential for lymphocyte activation and proliferation,^7^ but lymphocytes lack *Slc7a11* expression and have a low cystine uptake capacity.^26,27^ Therefore, other cell types must support lymphocytes for glutathione synthesis. *In vitro*, dendritic cells and fibroblasts expressing *Slc7a11* contribute to extracellular redox regulation through glutathione synthesis and maintain cysteine supply to *Slc7a11*-deficient cells, such as lymphocytes.^14,21–24^ However, it is unclear whether this mechanism contributes to the formation of TLS *in vivo*.

**Figure 4:**
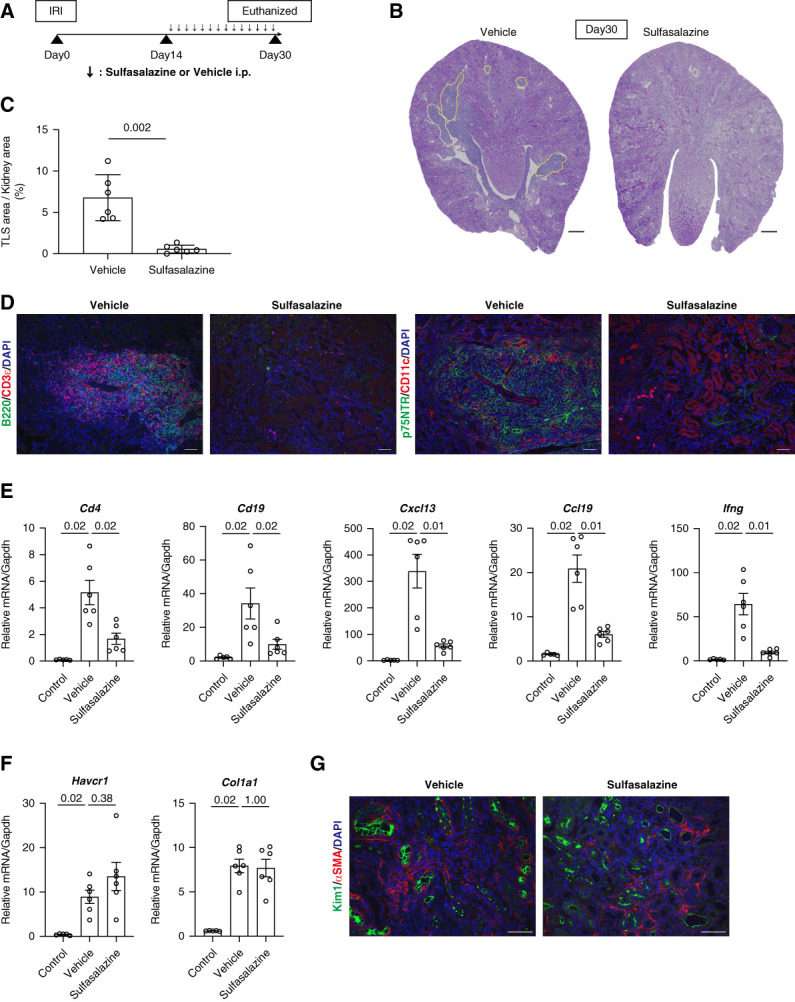

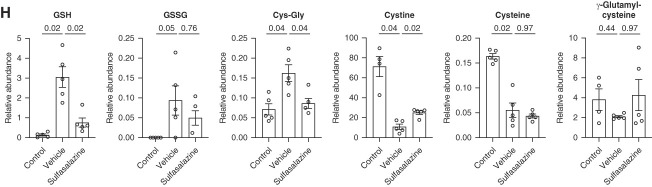
**Inhibition of the cystine/glutamate transporter prevents the formation of TLS in aged injured kidney.** (A) Experimental protocol for (B–H). (B) Representative images of the kidneys 30 days after IRI treated with sulfasalazine or vehicle from day 14 in PAS staining. TLS are encircled by yellow lines. (C) Cumulative sizes of TLS per kidney cortex area (*n*=6 in each group). (D) Immunofluorescence of B220 (green), CD3ε (red), and DAPI (blue) and p75NTR (green), CD11c (red), and DAPI (blue) in the kidneys 30 days after IRI treated with sulfasalazine or vehicle from day 14. (E) *Cd4*, *Cd19*, *Cxcl13*, *Ccl19*, and *Ifng* mRNA levels in the IRI kidneys (control: kidneys 30 days after sham operation, *n*=5; vehicle: kidneys 30 days after IRI treated by vehicle from day 14, *n*=6; sulfasalazine: the kidneys 30 days after IRI treated with sulfasalazine 400 mg/kg from day 14, *n*=6). (F) *Havcr1* and *Col1a1* mRNA levels in the IRI kidneys (control: kidneys 30 days after sham operation, *n*=5; vehicle: kidneys 30 days after IRI treated by vehicle from day 14, *n*=6; sulfasalazine: the kidneys 30 days after IRI treated with sulfasalazine 400 mg/kg from day 14, *n*=6). (G) Immunofluorescence of Kim1 (green), *α*SMA (red), and DAPI (blue) of the IRI kidneys treated with either vehicle or sulfasalazine. (H) Metabolome analysis of metabolites related to glutathione synthesis pathway (*n*=4 or 5 in each group). Values are means±SEM. Data were analyzed by (C) Mann–Whitney *U* test and (E, F, and H) Steel test with the vehicle-treated group as control. Scale bars: (B) 300 *µ*m; (D and G) 50 *µ*m. *α*SMA, alpha-smooth muscle actin; PAS, periodic acid–Schiff.

To investigate this, we pharmacologically inhibited the cystine/glutamate transporter in the model of TLS. Sulfasalazine, a potent inhibitor of the cystine/glutamate transporter, was administered daily from day 14 after IRI, when the formation of TLS was initiated (a preventive model; Figure 4A). Interestingly, the formation of TLS was significantly inhibited in the kidneys of sulfasalazine-treated mice (Figure 4, B–D). Sulfasalazine treatment also decreased the expression of *Cd4* (a T-cell marker) and *Cd19* (a B-cell marker), consistent with the suppression of the formation of TLS (Figure 4E). Furthermore, the expression of TLS-associated homeostatic chemokines and cytokine (*Cxcl13*, *Ccl19*, and *Ifng*) also decreased.

Although the formation of TLS was shown to correlate with kidney damage,^17^ *Havcr1* (the gene encoding Kim1) and *Col1a1* mRNA expression was not attenuated by sulfasalazine treatment, despite the suppression of TLS (Figure 4F). Immunofluorescence of Kim1 and *α*SMA also showed that the degree of tubular injury and interstitial fibrosis was similar in both groups (Figure 4G). This could be attributed to sulfasalazine-induced nephrotoxicity, as sulfasalazine is known to induce crystalluria and accumulation of reactive oxygen species in the kidney.^28,29^ Kim1 expression increased with higher doses of sulfasalazine (Supplemental Figure 6), suggesting a dose-dependent nephrotoxic effect. Similar to Kim1, desmin expression was observed in IRI kidneys from both sulfasalazine-treated and vehicle-treated mice, and in contralateral kidneys, its expression was more prominent in sulfasalazine-treated mice (Supplemental Figure 7). These results indicate that sulfasalazine induces tubulointerstitial injury and that inhibition of the formation of TLS by sulfasalazine is not mediated by the attenuation of kidney damage.

Metabolome analysis confirmed that sulfasalazine treatment decreased glutathione and cysteine-glycine levels in the kidney (Figure 4H). Although cyst(e)ine levels did not fully recover, presumably because of impaired cystine reabsorption due to sustained tubular injury by sulfasalazine,^28,29^ cystine levels tended to increase in the sulfasalazine group. This could be attributed to attenuated formation of TLS that consume cyst(e)ine to synthesize glutathione.

We also administered sulfasalazine daily from day 30, when TLS were already established, to day 45 post-IRI to assess whether it could revert established TLS (a treatment model; Supplemental Figure 8A). Although the effect was not as pronounced as in a preventive model in Figure 4, sulfasalazine treatment from day 30 partially reversed the formation of TLS (Supplemental Figure 8, B–D), with a corresponding decrease in the expression of TLS-related molecules (Supplemental Figure 8E). In addition, we assessed the effect of sulfasalazine in unilateral ureteral obstruction (UUO) model, another established model of the formation of TLS in the kidney (Supplemental Figure 9A). Although TLS were smaller in size compared with IRI model, they became evident by day 14 after UUO. The formation of TLS was significantly inhibited in the kidneys of sulfasalazine-treated mice (Supplemental Figure 9, B–D). The expression of *Cxcl13* and *Ifng* was reduced by sulfasalazine treatment, presumably because of the reduced size of TLS (Supplemental Figure 9E). Consistent with the findings in IRI model, GSH levels were reduced in the kidney of sulfasalazine-treated mice (Supplemental Figure 9F), presumably reflecting the attenuation of the formation of TLS. These findings indicate that sulfasalazine attenuates the formation of TLS in two independent models.

To further support the *in vivo* findings, we conducted *in vitro* coculture experiments that mimic the microenvironment of TLS (Supplemental Figure 10A). A previous report confirmed intercellular glutathione exchange between dendritic cells and lymphocytes and showed its inhibition by sulfasalazine^14^; to complement this, we used *Slc7a11*-specific siRNA (Supplemental Figure 10B). We found that intracellular glutathione levels in T cells cocultured with *Slc7a11*-knockdown (KD) dendritic cells significantly decreased under both normal conditions and oxidative stress induced by tert-butyl hydroperoxide (TBHP) (Supplemental Figure 10, C–E). By contrast, T cells cocultured with *Slc7a11*-KD fibroblasts did not show significant decrease in intracellular glutathione levels under normal conditions, but a decrease was observed under oxidative stress. Furthermore, the decrease of intracellular glutathione levels was also observed in T cells cocultured with dendritic cells or fibroblasts pretreated with sulfasalazine (Supplemental Figure 11). These findings suggest that intercellular glutathione exchange occurs between lymphocytes and dendritic cells or fibroblasts and plays an important role under oxidative stress conditions, as seen in TLS.

**Figure 5:**
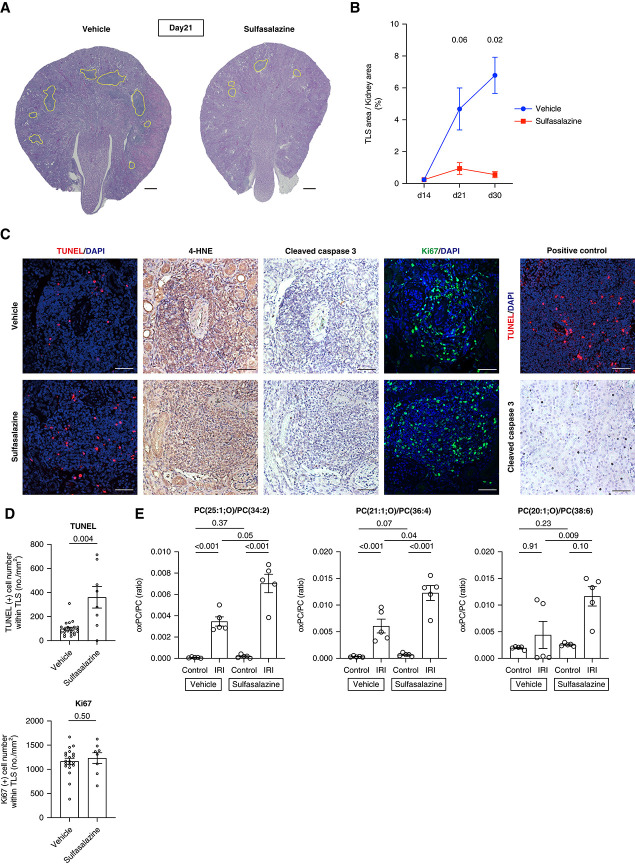
**Oxidized lipids that trigger ferroptosis accumulate in sulfasalazine-treated kidneys after IRI.** (A) Representative images of the kidneys 21 days after IRI treated with sulfasalazine or vehicle from day 14 in PAS staining. Sulfasalazine or vehicle was administered in the same protocol as in Figure 4A. TLS are encircled by the yellow lines. (B) Quantification of the size of TLS in the kidneys at day 14 (*n*=5), day 21 (*n*=5), and day 30 after IRI (*n*=6 in each group). (C) TUNEL staining (red) with DAPI staining (blue), immunohistochemistry of 4-HNE and cleaved caspase 3, and immunofluorescence for Ki67. Samples of the healthy spleens and kidneys of the cisplatin nephropathy model were used as positive controls for TUNEL staining and immunohistochemistry of cleaved caspase 3, respectively. 4-HNE and cleaved caspase 3 were detected by DAB staining. (D) Numbers of TUNEL-positive cells and Ki67-positive cells within TLS per the size of TLS in the kidneys treated with sulfasalazine or vehicle 21 days after IRI. (E) Ratio of OxPCs per PCs in the kidneys treated with sulfasalazine or vehicle 21 days after IRI. Contralateral kidneys in each group were used as control (*n*=5 in each group). PC (25:1;O), PC (21:1;O), and PC (20:1;O) are oxidized form of linoleic acid in PC (34:2), arachidonic acid in PC (36:4), and docosahexaenoic acid in PC (38:6), respectively. Values are means±SEM. Data were analyzed by (B) multiple Mann–Whitney *U* test, (D) Mann–Whitney *U* test, and (E) two-way ANOVA after aligned rank transform. Scale bars: (A) 300 *µ*m; (C) 50 *µ*m. OxPC, oxidized phosphatidylcholine; TUNEL, terminal deoxynucleotidyl transferase–mediated dUTP nick-end labeling.

The inhibition of the cystine/glutamate transporter hinders glutathione synthesis and induces ferroptosis, a regulated form of cell death characterized by excessive accumulation of lipid peroxides.^30,31^ Considering the significant reduction in TLS on day 30 post-IRI, we investigated oxidized lipids within the kidneys on day 21 post-IRI, a timepoint corresponding to active formation of TLS. The formation of TLS was suppressed in the kidneys of sulfasalazine-treated mice on day 21, although still observed (Figure 5, A and B, Supplemental Figure 12). TUNEL-positive cells and 4-HNE accumulation were observed within TLS in both sulfasalazine-treated and vehicle-treated mice, while cleaved caspase-3, an apoptosis marker, was not evident in either group (Figure 5C). TUNEL-positive cells within TLS increased in the sulfasalazine-treated group, while the number of Ki67-positive cells remained unchanged (Figure 5, C and D). These findings indicate that nonapoptotic cell death might occur within TLS and be further expedited by the inhibition of the cystine/glutamate transporter. The ratio of oxidized phosphatidylcholines (OxPCs) to nonoxidized phosphatidylcholines (phosphatidylcholines [PCs]; the most abundant phospholipid in mammalian cells^32^) was elevated in the kidneys of vehicle-treated mice (Figure 5E). Interestingly, OxPCs accumulated to a greater extent in the kidneys of sulfasalazine-treated mice. In particular, higher levels of PC (25:1;O; 1-palmitoyl-2-(9′-oxo-nonanoyl)-sn-glycero-3-phosphocholine) and PC (21:1;O; 1-palmitoyl-2-(5′-oxo-valeroyl)-sn-glycero-3-phosphocholine), heavily toxic OxPCs that trigger ferroptosis,^33–35^ accumulated in sulfasalazine-treated kidneys after IRI. We also examined the expression of key ferroptosis-related genes: *Gpx4* and *Acsl4*. In the kidneys with TLS, *Gpx4* expression was modestly decreased, while *Acsl4* expression was increased compared with controls, consistent with ferroptosis-associated changes (Supplemental Figure 13). After sulfasalazine treatment, *Gpx4* expression showed a slight increase toward baseline levels, whereas *Acsl4* expression remained elevated. Although further validation is required, these results may suggest that the inhibition of the cystine/glutamate transporter could disrupt the equilibrium between cell proliferation and cell death (possibly ferroptosis) within TLS, leading to attenuation of TLS.

### Urinary GSH Was a Candidate Biomarker for Tertiary Lymphoid Structures in Mice and Patients with IgA Nephropathy

**Figure 6:**
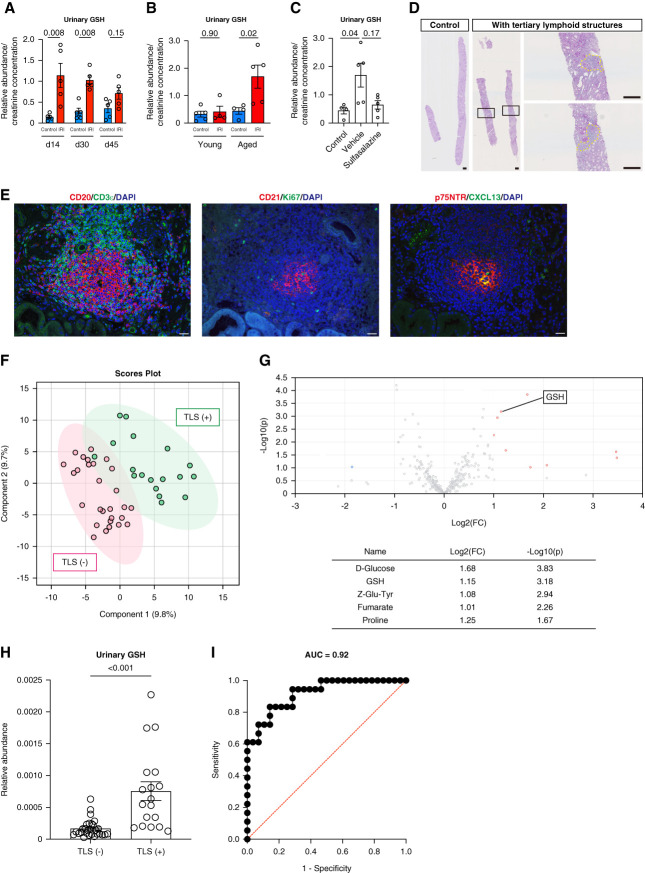
**Urinary GSH concentrations as a potential biomarker for the presence of TLS in the kidney in mice and patients with IgA nephropathy.** (A) Urinary GSH levels at indicated timepoints after IRI and sham surgeries in aged mice (*n*=5 in each group). (B) Urinary GSH levels in young and aged mice at day 30 after IRI and sham surgeries (*n*=5 in each group). (C) Urinary GSH levels of mice 30 days after IRI, treated with either sulfasalazine or vehicle in the model illustrated in Figure 4 (control: mice 30 days post-sham operation, *n*=4; vehicle: mice 30 days post-IRI, treated with vehicle from day 14, *n*=5; sulfasalazine: mice 30 days post-IRI, treated with 400 mg/kg sulfasalazine from day 14, *n*=5). (D) Representative images of PAS staining of human kidney biopsy samples with and without TLS from IgA nephropathy patients. The regions delineated by square areas are enlarged for detailed demonstration. TLS are encircled by dotted lines. (E) Immunofluorescence of CD20 (red), CD3ε (green), and DAPI (blue); CD21 (red), Ki67 (green), and DAPI (blue); and p75NTR (green), CXCL13 (red), and DAPI (blue) in human kidneys with TLS. (F) Principal component plot to represent the clustering patterns of metabolite profiles in urine samples from IgA nephropathy patients with TLS (+) and without TLS (‒) in the kidneys. Each point corresponds to a unique urine sample, and the distance between points reflects the similarity of metabolite compositions. Green and red dots represent urine samples from patients with and without TLS, respectively. (G) Volcano plot of metabolite profiles of urine samples from IgA nephropathy patients. Each point on the plot represents a metabolite, with the *x* axis indicating fold change and the *y* axis indicating statistical significance. Upregulated and downregulated metabolites with −Log10(*P*) > 1 and Log2(FC)>1 are highlighted in red and blue, respectively. The five metabolites exhibiting the most pronounced statistical significance are listed. (H) GSH levels in urine samples of IgA nephropathy patients with and without TLS in the kidney (*n*=18 and 28, respectively). (I) ROC curve to illustrate the performance of the logistic regression model, as presented in Table 2. The solid line connecting dots represents the model's discrimination, with an AUC of 0.92. GSH levels were measured by mass spectrometry and normalized by the creatinine concentration. Values are means±SEM. Data were analyzed by (A, B, and H) Mann–Whitney *U* test and (C) Steel test with the vehicle-treated group as control. Scale bars: (D) 250 *µ*m; (E) 20 *µ*m. AUC, area under the curve; ROC, receiver operating characteristic.

Next, we examined whether glutathione accumulation within TLS is reflected in urinary GSH concentrations. Indeed, urinary GSH levels increased over the course of the formation of TLS after IRI (Figure 6A). On the contrary, serum GSH levels did not differ significantly between mice with TLS and control mice (Supplemental Figure 14), suggesting that increased urinary GSH concentrations are not simply a reflection of elevated serum GSH levels. In addition, we compared urinary GSH levels in aged mice with TLS and in young mice after the same extent of IRI, which lack TLS in the kidney, as previously demonstrated.^11^ Unlike aged mice, urinary GSH levels in young mice did not significantly increase, despite both groups experiencing the same extent of IRI (Figure 6B). This indicates that the upregulation of glutathione synthesis within TLS is possibly reflected by increased urinary GSH concentrations. Urinary GSH levels also tended to decrease after sulfasalazine treatment, possibly due to the reduction of TLS (Figures 4 and 6C).

Next, we comprehensively investigated the metabolites in urine samples from 46 patients with IgA nephropathy (Table 1 and Supplemental Figure 1) by nontargeted metabolome analysis. The presence or absence of TLS in the kidney was confirmed by periodic acid–Schiff and immunofluorescence staining (Figure 6, D and E). Similar to murine TLS and human TLS in nephrectomized kidneys (Figure 2), 4-HNE and 8-OHdG accumulation was also observed within TLS of IgA nephropathy patients (Supplemental Figure 15). Multivariable analysis (partial least squares-discriminant analysis) revealed that urinary metabolite profiles differed depending on the presence or absence of TLS in the kidney, and GSH was one of the metabolites that exhibited the most significant alterations (Figure 6, F and G). Indeed, urinary GSH levels were significantly elevated in patients with TLS in the kidney (Figure 6H). A multivariable logistic regression model was constructed to estimate the presence of TLS in the kidney with two covariables: urinary GSH levels and eGFR (Table 2). This model showed high accuracy in estimating the presence of TLS in the kidney, and the receiver operating characteristic curve revealed a high area under the curve value of 0.92 (Figure 6I). The optimal sensitivity and specificity were 86% and 83%, respectively. These results suggest that similar metabolic alterations occur in murine and human kidneys with TLS and that urinary GSH could be a noninvasive diagnostic biomarker to detect TLS in the kidney.

**Table 2:** Multivariable logistic regression analysis of determinants associated with tertiary lymphoid structures in the kidney

Two Variables	OR (95% CI)
Urinary GSH	13.72 (1.35 to 139.23)
eGFR	0.28 (0.07 to 1.07)

All variables are standardized and redefined as variables/SD. CI, confidence interval; GSH, glutathione; OR, odds ratio.

In addition, we analyzed urinary GSH levels in patients with TLS in the kidney at the time of diagnosis and 1 year later. Of the 18 patients with TLS in the kidney recruited in Figure 6, nine received steroid treatment and had urine samples available at both timepoints (Supplemental Table 1). While overall urinary GSH levels before and after the treatment did not show a significant difference (Supplemental Figure 16A), a significant decrease in urinary GSH levels was observed in seven patients who experienced more than a 50% reduction in urinary protein levels after steroid treatment (Supplemental Figure 16B). Although we were unable to confirm the regression of TLS due to the lack of rebiopsy, together with the previous reports indicating that steroid treatment reduces the size of TLS^17^ and that the presence of TLS is correlated with increased proteinuria in IgA nephropathy patients,^2^ these findings possibly suggest that urinary GSH levels may reflect the resolution of TLS in certain subpopulations of IgA nephropathy patients.

## Discussion

There is a lack of knowledge about alterations of the metabolic system that supports the formation of TLS. In this study, we revealed that active glutathione synthesis contributes to the formation of TLS. We further demonstrated that an increase in urinary GSH concentrations during the formation of TLS is a common phenomenon in both mice and humans and proposed the utility of urinary GSH as a biomarker to detect TLS in the kidney.

Lymphocyte proliferation and immune responses require vast metabolic resources, especially thiols such as cysteine and glutathione.^7,8^ Although the rate of glutathione synthesis depends on cysteine availability, cysteine is quickly oxidized to its dimer form cystine in the extracellular space (Figure 1E). Indeed, cysteine concentrations in serum and culture medium are very low compared with that of cystine.^36,37^ Therefore, cells that require glutathione for their survival and proliferation must import cystine from the extracellular environment through the cystine/glutamate transporter (encoded by *Slc7a11*). The expression of the cystine/glutamate transporter is induced in the context of high oxidative stress and cell proliferation,^15,38,39^ and its inhibition hinders glutathione synthesis leading to ferroptosis.^30,31,40,41^ In immune cells, T cells do not express the cystine/glutamate transporter and depend instead on thiol supply from other cell types for their survival *in vitro*.^14,24,26,42,43^ However, it was unclear whether the intercellular glutathione exchange shown *in vitro* also occurs *in vivo*.

Dendritic cells and fibroblasts play important roles in the formation of TLS.^11,44^ Dendritic cells express the cystine/glutamate transporter, import cystine, and export synthesized glutathione to the extracellular space through MRP1.^14,21,24^ Thereby, dendritic cells maintain the surrounding environment in a reducing state and support lymphocyte proliferation.^14,21^ Fibroblasts also import cystine through the cystine/glutamate transporter and reduce it to cysteine, maintaining the pericellular environment in a reducing state *in vitro*.^22,23^ In this study, we found that dendritic cells and fibroblasts within TLS in the kidney exclusively express the cystine/glutamate transporter and that glutathione supply from these cells to lymphocytes seems to be required for the formation of TLS and maintenance *in vivo*. Intercellular glutathione exchange between dendritic cells/fibroblasts and lymphocytes was also suggested by our coculture experiment (Supplemental Figures 10 and 11), although we acknowledge that our *in vitro* experiments do not fully recapitulate the *in vivo* phenomena. Although the number of *Slc7a11*-expressing dendritic cells or fibroblasts within TLS is limited (Supplemental Figure 5) and the mechanism by which this small population contributes to the substantial glutathione accumulation within TLS (Figure 1) remains unclear, our findings suggest that *Slc7a11*-expressing dendritic cells and fibroblasts play a pivotal role in driving glutathione synthesis within the microenvironment of TLS.

Sulfasalazine is a potent inhibitor of the cystine/glutamate transporter that inhibits glutathione synthesis and induces ferroptosis^30,31^ and is regarded as a promising medication for cancer.^16,41,45–47^ Although there are several inhibitors of the cystine/glutamate transporter other than sulfasalazine, these drugs have limitations of low bioavailability and low selectivity to of the cystine/glutamate transporter *in vivo*.^48,49^ In our study, sulfasalazine administration inhibited the formation of TLS and attenuated the expression of TLS-related cytokines, with sustained tubular injury. Although these findings do not completely exclude the effect on kidney parenchymal cells or off-target effects, these results suggest that sulfasalazine prevents the formation of TLS not by attenuating tubular injury but *via* its inhibitory effect on of the cystine/glutamate transporter specifically expressed within TLS. Although the direct application of sulfasalazine in patients with kidney diseases is difficult because of its nephrotoxicity,^28,29^ the inhibition of specific metabolic pathways of TLS may represent a new therapeutic approach against kidney diseases.

TLS in the kidney are associated with aggravated histologic inflammation and impaired kidney function, representing novel therapeutic targets in kidney diseases.^6^ We previously revealed that the stage and number of TLS correlate with the extent of tissue damage in both mice and humans, and prognosis of kidney transplants.^4,17^ Another group showed that the presence of TLS correlates with tissue damage and poor prognosis in patients with IgA nephropathy.^2^ Therefore, early detection of TLS in the kidney could identify patients who would benefit from prompt treatment. As demonstrated in this study, urinary GSH levels accurately estimated the presence of TLS in the kidney in patients with IgA nephropathy. Thus, urinary GSH may have important clinical implications as a promising biomarker for identifying TLS in the kidney.

Our study has some limitations. First, we could not perform imaging mass spectrometry in human samples because of the need for unfixed, immediately frozen tissue samples. Second, RNAscope of *Slc7a11* in human kidney samples was unsuccessful, probably because of the sample fixation conditions. Third, since conditional *Slc7a11* knockout mice were unavailable, our *in vivo* analysis relied on pharmacologic inhibition of the cystine/glutamate transporter and cannot completely exclude off-target effects of sulfasalazine or the contribution of other cell types beyond dendritic cells and fibroblasts in glutathione synthesis.

Despite these limitations, our study revealed the unique metabolic microenvironment of TLS in the kidney (Supplemental Figure 17). Glutathione synthesis plays key roles in the formation of TLS, and a specific metabolic pathway in a limited cell population within TLS determines the outcome of the formation of TLS. Furthermore, elevated glutathione synthesis in TLS in the kidney is reflected in urinary GSH concentration, suggesting the possibility of its clinical application as a novel biomarker to detect TLS in the kidney. Future studies will further elucidate the currently unrecognized metabolic landscape of TLS.

## Supplementary Material

## Data Availability

This study includes clinical experimentation and received Institutional Review Board or Ethics Committee approval. All patients provided written informed consent. This study includes clinical experimentation and complies with the Declaration of Helsinki. All animal experiments were conducted in accordance with the NIH Guide for the Care and Use of Laboratory Animals or an equivalent standard that meets or exceeds the ethical and welfare requirements outlined in the NIH Guide. All protocols were approved by the appropriate institutional animal care and use committee.

## Data Availability Statements

Original data generated for the study will be made available on reasonable request to the corresponding author. Data Type: Raw Data/Source Data; Research Protocols. Although the dataset regarding patient analysis is not available because sharing of individual participant data with third parties was not specifically included in the informed consent of the study, other data are available from the authors on reasonable request and with permission of the ethical committee of Kyoto University Graduate School and Faculty of Medicine.

## Supplemental Material

Supplemental Figure 1. Enrollment for histologic evaluation of TLS in the kidney and metabolome analysis of urine samples.

Supplemental Figure 2. Desorption electrospray ionization–imaging mass spectrometry of GSH in the kidney with TLS.

Supplemental Figure 3. PPP is upregulated in TLS in the kidney.

Supplemental Figure 4. Higher oxidative stress accumulates in immune cells within TLS compared with those within the spleen and peripheral blood.

Supplemental Figure 5. The percentages of *Slc7a11* (+) MRP1 (+) dendritic cells and fibroblasts within TLS.

Supplemental Figure 6. Tubular injury in the kidney following different dosages of sulfasalazine treatment.

Supplemental Figure 7. Desmin expression in the kidney of mice treated with sulfasalazine or vehicle after IRI.

Supplemental Figure 8. Sulfasalazine treatment contributes to partial reversion of TLS in the kidney.

Supplemental Figure 9. Sulfasalazine treatment prevents the formation of TLS in UUO model.

Supplemental Figure 10. Intracellular glutathione levels significantly decrease in T cells cocultured with *Slc7a11*-knockdown dendritic cells or fibroblasts.

Supplemental Figure 11. Intracellular glutathione levels of T cells cocultured with dendritic cells or fibroblasts pretreated with sulfasalazine.

Supplemental Figure 12. Time course immunostaining of the kidneys treated with sulfasalazine or vehicle after IRI.

Supplemental Figure 13. Alterations of Gpx4 and Acsl4 expression after sulfasalazine treatment.

Supplemental Figure 14. Serum GSH concentrations do not increase in mice with TLS in the kidney.

Supplemental Figure 15. 8-OHdG and 4-HNE accumulate within TLS in the kidney of IgA nephropathy patients.

Supplemental Figure 16. Urinary GSH concentrations at baseline and 1 year after diagnosis in IgA nephropathy patients with TLS in the kidney.

Supplemental Figure 17. A scheme showing metabolic microenvironment of TLS and clinical potential of urinary glutathione as a biomarker to detect TLS in the kidney.

Supplemental Table 1. Changes in serum creatinine, eGFR, and urinary protein levels in IgA nephropathy patients at baseline and 1 year after kidney biopsy.

Supplemental Table 2. Primer sequences used for real-time PCR.
