# Supplementary material for: Glutathione Synthesis via the Cystine/Glutamate Transporter Promotes the Formation of Tertiary Lymphoid Structures in the Kidney
Source: J Am Soc Nephrol. 2025 Aug 8;37(2):283–98. doi: 10.1681/ASN.0000000825 (PMC12889938; doi:10.1681/ASN.0000000825)
Supplement: Supplementary file 1 [file jasn-37-283-s001.pdf]

ASN American Society of Nephrology | JASN Journal of the American Society of Nephrology | KIDNEY360 Accessing Our World From Every Angle | CJASN Clinical Journal of the American Society of Nephrology

ASN Journal Disclosure Form

As per ASN journal policy, I have disclosed any financial relationships or commitments I have held in the past 36 months as included below. I have listed my Current Employer below to indicate there is a relationship requiring disclosure. If no relationship exists, my Current Employer is not listed.

H. Arai reports the following:
Employer: Kyoto University

I understand that the information above will be published within the journal article, if accepted, and that failure to comply and/or to accurately and completely report the potential financial conflicts of interest could lead to the following: 1) Prior to publication, article rejection, or 2) Post-publication, sanctions ranging from, but not limited to, issuing a correction, reporting the inaccurate information to the authors' institution, banning authors from submitting work to ASN journals for varying lengths of time, and/or retraction of the published work.

Name: Hiroyuki Arai
Manuscript ID: JASN-2025-000331R1
Manuscript Title: Glutathione synthesis via the cystine/glutamate transporter promotes the formation of tertiary lymphoid structures in the kidney
Date of Completion: July 4, 2025
Disclosure Updated Date: July 4, 2025

ASN American Society of Nephrology | JASN Journal of the American Society of Nephrology | KIDNEY360 Accessing Our World From Every Angle | CJASN Clinical Journal of the American Society of Nephrology

ASN Journal Disclosure Form

As per ASN journal policy, I have disclosed any financial relationships or commitments I have held in the past 36 months as included below. I have listed my Current Employer below to indicate there is a relationship requiring disclosure. If no relationship exists, my Current Employer is not listed.

S. Fukuma reports the following:
Employer: Kyoto University, Hiroshima University; Consultancy: Kyowa Hakko Kirin, Rege Nephro, White Healthcare; Research Funding: Kyowa Hakko Kirin, CANCER SCAN; Honoraria: Kyowa Hakko Kirin, Nippon Boehringer Ingelheim; Advisory or Leadership Role: Kyowa Hakko Kirin; and Speakers Bureau: Japan Boehringer Ingelheim, Kyowa Kirin, Sanwa Chemical.

I understand that the information above will be published within the journal article, if accepted, and that failure to comply and/or to accurately and completely report the potential financial conflicts of interest could lead to the following: 1) Prior to publication, article rejection, or 2) Post-publication, sanctions ranging from, but not limited to, issuing a correction, reporting the inaccurate information to the authors' institution, banning authors from submitting work to ASN journals for varying lengths of time, and/or retraction of the published work.

Name: Shingo Fukuma
Manuscript ID: JASN-2025-000331R1
Manuscript Title: Glutathione synthesis via the cystine/glutamate transporter promotes the formation of tertiary lymphoid structures in the kidney
Date of Completion: July 9, 2025
Disclosure Updated Date: July 6, 2025

ASN Journal Disclosure Form

As per ASN journal policy, I have disclosed any financial relationships or commitments I have held in the past 36 months as included below. I have listed my Current Employer below to indicate there is a relationship requiring disclosure. If no relationship exists, my Current Employer is not listed.

S. Goto reports the following:
Employer: Sumitomo Pharma Co., Ltd.

I understand that the information above will be published within the journal article, if accepted, and that failure to comply and/or to accurately and completely report the potential financial conflicts of interest could lead to the following: 1) Prior to publication, article rejection, or 2) Post-publication, sanctions ranging from, but not limited to, issuing a correction, reporting the inaccurate information to the authors' institution, banning authors from submitting work to ASN journals for varying lengths of time, and/or retraction of the published work.

Name: Shima Goto
Manuscript ID: JASN-2025-000331R1
Manuscript Title: Glutathione synthesis via the cystine/glutamate transporter promotes the formation of tertiary lymphoid structures in the kidney
Date of Completion: July 5, 2025
Disclosure Updated Date: July 5, 2025

ASN
American Society of Nephrology
JASN
Journal of the American Society of Nephrology
KIDNEY360
Accessing Our World From Every Angle
CJASN
Clinical Journal of the American Society of Nephrology

ASN Journal Disclosure Form

As per ASN journal policy, I have disclosed any financial relationships or commitments I have held in the past 36 months as included below. I have listed my Current Employer below to indicate there is a relationship requiring disclosure. If no relationship exists, my Current Employer is not listed.

R. Kamimatsuse reports the following:
Employer: Kyoto University Hospital

I understand that the information above will be published within the journal article, if accepted, and that failure to comply and/or to accurately and completely report the potential financial conflicts of interest could lead to the following: 1) Prior to publication, article rejection, or 2) Post-publication, sanctions ranging from, but not limited to, issuing a correction, reporting the inaccurate information to the authors' institution, banning authors from submitting work to ASN journals for varying lengths of time, and/or retraction of the published work.

Name: Ryo Kamimatsuse
Manuscript ID: JASN-2025-000331R1
Manuscript Title: Glutathione synthesis via the cystine/glutamate transporter promotes the formation of tertiary lymphoid structures in the kidney
Date of Completion: July 7, 2025
Disclosure Updated Date: July 7, 2025

ASN American Society of Nephrology | JASN Journal of the American Society of Nephrology | KIDNEY360 Accessing Our World From Every Angle | CJASN Clinical Journal of the American Society of Nephrology

ASN Journal Disclosure Form

As per ASN journal policy, I have disclosed any financial relationships or commitments I have held in the past 36 months as included below. I have listed my Current Employer below to indicate there is a relationship requiring disclosure. If no relationship exists, my Current Employer is not listed.

M. Kondo has nothing to disclose.

I understand that the information above will be published within the journal article, if accepted, and that failure to comply and/or to accurately and completely report the potential financial conflicts of interest could lead to the following: 1) Prior to publication, article rejection, or 2) Post-publication, sanctions ranging from, but not limited to, issuing a correction, reporting the inaccurate information to the authors' institution, banning authors from submitting work to ASN journals for varying lengths of time, and/or retraction of the published work.

Name: Makiko Kondo
Manuscript ID: JASN-2025-000331R1
Manuscript Title: Glutathione synthesis via the cystine/glutamate transporter promotes the formation of tertiary lymphoid structures in the kidney
Date of Completion: July 7, 2025
Disclosure Updated Date: July 7, 2025

ASN Journal Disclosure Form

As per ASN journal policy, I have disclosed any financial relationships or commitments I have held in the past 36 months as included below. I have listed my Current Employer below to indicate there is a relationship requiring disclosure. If no relationship exists, my Current Employer is not listed.

R. Maeda reports the following:
Employer: Kyoto University

I understand that the information above will be published within the journal article, if accepted, and that failure to comply and/or to accurately and completely report the potential financial conflicts of interest could lead to the following: 1) Prior to publication, article rejection, or 2) Post-publication, sanctions ranging from, but not limited to, issuing a correction, reporting the inaccurate information to the authors' institution, banning authors from submitting work to ASN journals for varying lengths of time, and/or retraction of the published work.

Name: Rae Maeda
Manuscript ID: JASN-2025-000331R1
Manuscript Title: Glutathione synthesis via the cystine/glutamate transporter promotes the formation of tertiary lymphoid structures in the kidney,
Date of Completion: July 13, 2025
Disclosure Updated Date: July 13, 2025

ASN Journal Disclosure Form

As per ASN journal policy, I have disclosed any financial relationships or commitments I have held in the past 36 months as included below. I have listed my Current Employer below to indicate there is a relationship requiring disclosure. If no relationship exists, my Current Employer is not listed.

Y. Matsuoka reports the following:
Employer: Kyoto University

I understand that the information above will be published within the journal article, if accepted, and that failure to comply and/or to accurately and completely report the potential financial conflicts of interest could lead to the following: 1) Prior to publication, article rejection, or 2) Post-publication, sanctions ranging from, but not limited to, issuing a correction, reporting the inaccurate information to the authors' institution, banning authors from submitting work to ASN journals for varying lengths of time, and/or retraction of the published work.

Name: Yuta Matsuoka
Manuscript ID: JASN-2025-000331R1
Manuscript Title: Glutathione synthesis via the cystine/glutamate transporter promotes the formation of tertiary lymphoid structures in the kidney
Date of Completion: July 6, 2025
Disclosure Updated Date: July 6, 2025

ASN American Society of Nephrology JASN Journal of the American Society of Nephrology KIDNEY360 Accessing Our World From Every Angle CJASN Clinical Journal of the American Society of Nephrology

ASN Journal Disclosure Form

As per ASN journal policy, I have disclosed any financial relationships or commitments I have held in the past 36 months as included below. I have listed my Current Employer below to indicate there is a relationship requiring disclosure. If no relationship exists, my Current Employer is not listed.

H. Neyama has nothing to disclose.

I understand that the information above will be published within the journal article, if accepted, and that failure to comply and/or to accurately and completely report the potential financial conflicts of interest could lead to the following: 1) Prior to publication, article rejection, or 2) Post-publication, sanctions ranging from, but not limited to, issuing a correction, reporting the inaccurate information to the authors' institution, banning authors from submitting work to ASN journals for varying lengths of time, and/or retraction of the published work.

Name: Hiroyuki Neyama
Manuscript ID: JASN-2025-000331R1
Manuscript Title: Glutathione synthesis via the cystine/glutamate transporter promotes the formation of tertiary lymphoid structures in the kidney
Date of Completion: July 12, 2025
Disclosure Updated Date: July 12, 2025

ASN Journal Disclosure Form

As per ASN journal policy, I have disclosed any financial relationships or commitments I have held in the past 36 months as included below. I have listed my Current Employer below to indicate there is a relationship requiring disclosure. If no relationship exists, my Current Employer is not listed.

Y. Sato has nothing to disclose.

I understand that the information above will be published within the journal article, if accepted, and that failure to comply and/or to accurately and completely report the potential financial conflicts of interest could lead to the following: 1) Prior to publication, article rejection, or 2) Post-publication, sanctions ranging from, but not limited to, issuing a correction, reporting the inaccurate information to the authors' institution, banning authors from submitting work to ASN journals for varying lengths of time, and/or retraction of the published work.

Name: Yuki Sato
Manuscript ID: JASN-2025-000331R1
Manuscript Title: Glutathione synthesis via the cystine/glutamate transporter promotes the formation of tertiary lymphoid structures in the kidney
Date of Completion: July 12, 2025
Disclosure Updated Date: July 12, 2025

ASN American Society of Nephrology JASN Journal of the American Society of Nephrology KIDNEY360 Accessing Our World From Every Angle CJASN Clinical Journal of the American Society of Nephrology

ASN Journal Disclosure Form

As per ASN journal policy, I have disclosed any financial relationships or commitments I have held in the past 36 months as included below. I have listed my Current Employer below to indicate there is a relationship requiring disclosure. If no relationship exists, my Current Employer is not listed.

Y. Sugiura reports the following:
Employer: Kyoto University

I understand that the information above will be published within the journal article, if accepted, and that failure to comply and/or to accurately and completely report the potential financial conflicts of interest could lead to the following: 1) Prior to publication, article rejection, or 2) Post-publication, sanctions ranging from, but not limited to, issuing a correction, reporting the inaccurate information to the authors' institution, banning authors from submitting work to ASN journals for varying lengths of time, and/or retraction of the published work.

Name: Yuki Sugiura
Manuscript ID: JASN-2025-000331R1
Manuscript Title: Glutathione synthesis via the cystine/glutamate transporter promotes the formation of tertiary lymphoid structures in the kidney,
Date of Completion: July 7, 2025
Disclosure Updated Date: July 7, 2025

ASN American Society of Nephrology JASN Journal of the American Society of Nephrology KIDNEY360 Accessing Our World From Every Angle CJASN Clinical Journal of the American Society of Nephrology

ASN Journal Disclosure Form

As per ASN journal policy, I have disclosed any financial relationships or commitments I have held in the past 36 months as included below. I have listed my Current Employer below to indicate there is a relationship requiring disclosure. If no relationship exists, my Current Employer is not listed.

K. Taniguchi reports the following:
Employer: Kyoto Univesiry; and Research Funding: Boehringer Ingelheim.

I understand that the information above will be published within the journal article, if accepted, and that failure to comply and/or to accurately and completely report the potential financial conflicts of interest could lead to the following: 1) Prior to publication, article rejection, or 2) Post-publication, sanctions ranging from, but not limited to, issuing a correction, reporting the inaccurate information to the authors' institution, banning authors from submitting work to ASN journals for varying lengths of time, and/or retraction of the published work.

Name: Keisuke Taniguchi
Manuscript ID: JASN-2025-000331R1
Manuscript Title: Glutathione synthesis via the cystine/glutamate transporter promotes the formation of tertiary lymphoid structures in the kidney
Date of Completion: July 7, 2025
Disclosure Updated Date: July 7, 2025

ASN American Society of Nephrology
JASN Journal of the American Society of Nephrology
KIDNEY360 Accessing Our World From Every Angle
CJASN Clinical Journal of the American Society of Nephrology

ASN Journal Disclosure Form

As per ASN journal policy, I have disclosed any financial relationships or commitments I have held in the past 36 months as included below. I have listed my Current Employer below to indicate there is a relationship requiring disclosure. If no relationship exists, my Current Employer is not listed.

N. Toriu has nothing to disclose.

I understand that the information above will be published within the journal article, if accepted, and that failure to comply and/or to accurately and completely report the potential financial conflicts of interest could lead to the following: 1) Prior to publication, article rejection, or 2) Post-publication, sanctions ranging from, but not limited to, issuing a correction, reporting the inaccurate information to the authors' institution, banning authors from submitting work to ASN journals for varying lengths of time, and/or retraction of the published work.

Name: Naoya Toriu
Manuscript ID: JASN-2025-000331R1
Manuscript Title: Glutathione synthesis via the cystine/glutamate transporter promotes the formation of tertiary lymphoid structures in the kidney
Date of Completion: July 7, 2025
Disclosure Updated Date: July 7, 2025

ASN
American Society of Nephrology
JASN
Journal of the American Society of Nephrology
KIDNEY360
Accessing Our World From Every Angle
CJASN
Clinical Journal of the American Society of Nephrology

ASN Journal Disclosure Form

As per ASN journal policy, I have disclosed any financial relationships or commitments I have held in the past 36 months as included below. I have listed my Current Employer below to indicate there is a relationship requiring disclosure. If no relationship exists, my Current Employer is not listed.

S. Yamamoto reports the following:
Employer: Kyoto University Hospital

I understand that the information above will be published within the journal article, if accepted, and that failure to comply and/or to accurately and completely report the potential financial conflicts of interest could lead to the following: 1) Prior to publication, article rejection, or 2) Post-publication, sanctions ranging from, but not limited to, issuing a correction, reporting the inaccurate information to the authors' institution, banning authors from submitting work to ASN journals for varying lengths of time, and/or retraction of the published work.

Name: Shinya Yamamoto
Manuscript ID: JASN-2025-000331R1
Manuscript Title: Glutathione synthesis via the cystine/glutamate transporter promotes the formation of tertiary lymphoid structures in the kidney
Date of Completion: July 6, 2025
Disclosure Updated Date: July 6, 2025

ASN American Society of Nephrology
JASN Journal of the American Society of Nephrology
KIDNEY360 Accessing Our World From Every Angle
CJASN Clinical Journal of the American Society of Nephrology

ASN Journal Disclosure Form

As per ASN journal policy, I have disclosed any financial relationships or commitments I have held in the past 36 months as included below. I have listed my Current Employer below to indicate there is a relationship requiring disclosure. If no relationship exists, my Current Employer is not listed.

M. Yanagita reports the following:
Employer: Kyoto University Graduate School of Medicine; Research Funding: Tanabe Mitsubishi, Boehringer Ingelheim; Honoraria: Astellas, Kyowa Kirin, Chugai, and others for lecture honoraria; Advisory or Leadership Role: Astrazeneca; Novartis; Viatris; and Speakers Bureau: Astellas, Kyowa Kirin, AstraZeneca, and others for lecture honoraria.

I understand that the information above will be published within the journal article, if accepted, and that failure to comply and/or to accurately and completely report the potential financial conflicts of interest could lead to the following: 1) Prior to publication, article rejection, or 2) Post-publication, sanctions ranging from, but not limited to, issuing a correction, reporting the inaccurate information to the authors’ institution, banning authors from submitting work to ASN journals for varying lengths of time, and/or retraction of the published work.

Name: Motoko Yanagita
Manuscript ID: JASN-2025-000331R2
Manuscript Title: "Glutathione Synthesis via the Cystine/Glutamate Transporter Promotes the Formation of Tertiary Lymphoid Structures in the Kidney
Date of Completion: July 25, 2025
Disclosure Updated Date: July 25, 2025

ASN American Society of Nephrology JASN Journal of the American Society of Nephrology KIDNEY360 Accessing Our World From Every Angle CJASN Clinical Journal of the American Society of Nephrology

ASN Journal Disclosure Form

As per ASN journal policy, I have disclosed any financial relationships or commitments I have held in the past 36 months as included below. I have listed my Current Employer below to indicate there is a relationship requiring disclosure. If no relationship exists, my Current Employer is not listed.

T. Yoshikawa has nothing to disclose.

I understand that the information above will be published within the journal article, if accepted, and that failure to comply and/or to accurately and completely report the potential financial conflicts of interest could lead to the following: 1) Prior to publication, article rejection, or 2) Post-publication, sanctions ranging from, but not limited to, issuing a correction, reporting the inaccurate information to the authors' institution, banning authors from submitting work to ASN journals for varying lengths of time, and/or retraction of the published work.

Name: Takahisa Yoshikawa
Manuscript ID: JASN-2025-000331R2
Manuscript Title: Glutathione Synthesis via the Cystine/Glutamate Transporter Promotes the Formation of Tertiary Lymphoid Structures in the Kidney
Date of Completion: July 25, 2025
Disclosure Updated Date: July 7, 2025
