# Supplementary material for: Glutathione Synthesis via the Cystine/Glutamate Transporter Promotes the Formation of Tertiary Lymphoid Structures in the Kidney
Source: J Am Soc Nephrol. 2025 Aug 8;37(2):283–98. doi: 10.1681/ASN.0000000825 (PMC12889938; doi:10.1681/ASN.0000000825)
Supplement: Supplementary file 2 [file jasn-37-283-s002.pdf]

Supplemental Material

Glutathione synthesis via the cystine/glutamate transporter promotes the formation of tertiary lymphoid structures in the kidney

Hiroyuki Arai, Yuki Sugiura, Shinya Yamamoto, Takahisa Yoshikawa, Yuta Matsuoka, Rae Maeda, Hiroyuki Neyama, Ryo Kamimatsuse, Shima Goto, Keisuke Taniguchi, Naoya Toriu, Makiko Kondo, Shingo Fukuma, Motoko Yanagita

Corresponding author.

Motoko Yanagita: Department of Nephrology, Graduate School of Medicine, Kyoto University, Shogoin-Kawahara-cho 54, Sakyo-ku, Kyoto 606-8507, Japan.

E-mail: motoy@kuhp.kyoto-u.ac.jp, Tel: +81-75-751-3860, Fax: +81-75-751-3859

Supplemental Material Table of Contents

Supplemental Methods

Supplemental Figures 1-17

Supplemental Figure 1. Enrollment for histological evaluation of tertiary lymphoid structures in the kidney and metabolome analysis of urine samples.

Supplemental Figure 2: DESI- imaging mass spectrometry of GSH in the kidney with tertiary lymphoid structures.

Supplemental Figure 3. Pentose phosphate pathway is upregulated in tertiary lymphoid structures in the kidney.

Supplemental Figure 4. Higher oxidative stress accumulates in immune cells within tertiary lymphoid structures compared with those within the spleen and peripheral blood.

Supplemental Figure 5. The percentages of *Slc7a11* (+) MRP1 (+) dendritic cells and fibroblasts within tertiary lymphoid structures.

Supplemental Figure 6. Tubular injury in the kidney following different dosages of sulfasalazine-treatment.

Supplemental Figure 7. Desmin expression in the kidney of mice treated with sulfasalazine or vehicle after IRI.

Supplemental Figure 8. Sulfasalazine treatment contributes to partial reversion of tertiary lymphoid structures in the kidney.

Supplemental Figure 9. Sulfasalazine treatment prevents the formation of tertiary lymphoid structures in unilateral ureteral obstruction model.

Supplemental Figure 10. Intracellular glutathione levels significantly decrease in T cells co-cultured with *Slc7a11*-knockdown dendritic cells or fibroblasts.

Supplemental Figure 11. Intracellular glutathione levels of T cells co-cultured with dendritic cells

or fibroblasts pre-treated with sulfasalazine.

Supplemental Figure 12. Time course immunostaining of the kidneys treated with sulfasalazine or vehicle after IRI.

Supplemental Figure 13. Alterations of Gpx4 and Acsl4 expression following sulfasalazine treatment.

Supplemental Figure 14. Serum GSH concentrations do not increase in mice with tertiary lymphoid structures in the kidney.

Supplemental Figure 15. 8-OHdG and 4-HNE accumulate within tertiary lymphoid structures in the kidney of IgA nephropathy patients.

Supplemental Figure 16. Urinary GSH concentrations at baseline and one year after diagnosis in IgA nephropathy patients with tertiary lymphoid structures in the kidney.

Supplemental Figure 17. A scheme showing metabolic microenvironment of tertiary lymphoid structures and clinical potential of urinary glutathione as a biomarker to detect tertiary lymphoid structures in the kidney.

Supplemental Table 1 and 2

Supplemental table 1: Changes in serum creatinine, eGFR, and urinary protein levels in IgA nephropathy patients at baseline and one year after renal biopsy.

Supplemental Table 2: Primer sequences used for real-time PCR

References for Supplemental Information

Supplemental Methods

Animals

We purchased 12-month-old C57BL6J male mice from Japan SLC. All mice were maintained under specific-pathogen-free conditions in the animal facility of Kyoto University. All animal experiments were approved by the Animal Research Committee, Graduate School of Kyoto University, and were performed in accordance with the *Guide for the Care and Use of Laboratory Animals* (National Institutes of Health, Bethesda, MD, USA). For generating P0-Cre/tdTomato mice, transgenic mice expressing Cre under the control of the P0 promoter (P0-Cre mice)[1] were mated with R26 tdTomato mice[2] to obtain double-transgenic mice.

Kidney injury models

Unilateral ischemia–reperfusion injury (IRI) was induced as described previously.[3] Briefly, mice were maintained at 37°C under anesthesia with 2% isoflurane inhalation. The left kidney was exteriorized through a small incision. IRI was induced by clamping the unilateral renal pedicles for 45 min. Adenine nephropathy was induced by feeding a 0.20% adenine diet (Research Diets, New Brunswick, NJ, USA) as previously described.[4]

Compound administration

Sulfasalazine (catalog S0883; Sigma-Aldrich, Saint Louis, MO, USA) (400 mg/kg) was administered to the mice by intraperitoneal injection daily from day14 to day30 after IRI (n = 6). Fresh sulfasalazine solution was prepared every day in 10% DMSO and 90% sterile phosphate-buffered saline.

Renal histochemistry

Harvested kidney samples were fixed in 10% formalin solution, embedded in paraffin, sectioned

(2.0 μm), and stained with periodic acid–Schiff (PAS). All PAS-stained samples were analyzed using a Keyence BZ-X710 all-in-one microscope and Zeiss Axio Imager A2 microscope.

Renal immunofluorescence

Immunofluorescence studies of mouse and human kidneys were performed as previously described.[5] The harvested kidney samples were fixed in 4% paraformaldehyde, incubated in 20% sucrose in PBS overnight, and incubated in 30% sucrose overnight at 4°C. OCT-embedded kidneys were cryosectioned into 6.0-μm sections. Formaldehyde-fixed and paraffin-embedded kidney samples were sectioned at a 4.0-μm thickness and used for immunofluorescence analysis. The sections were deparaffinized with xylene and rehydrated with ethanol. Antigen retrieval at 15 minutes at 110°C with the citrate buffer or 10 minutes at 95°C with Dako Target Retrieval Solution, pH 9 (catalog S2367; Dako, Glostrup, Denmark) was performed for deparaffinized and rehydrated sections. These sections were blocked with 5% serum appropriate for secondary antibodies for 1 hour at room temperature and then incubated with primary antibodies for overnight at 4°C. The following primary antibodies were used for immunostaining: anti-CD3ε (catalog ab5690; Abcam, Cambridge, UK), anti-CD20 (catalog 14-0202; eBioscience, San Diego, CA), anti-B220 (catalog 557390; BD PharMingen, San Diego, CA, USA), anti-CD11c (catalog 550283; BD PharMingen), anti-rBAT (catalog 16343-1-AP; Proteintech, Rosemont, IL, USA), anti-MRP1 (catalog ALX-801-007; Enzo Life Sciences, Farmingdale, NY, USA), anti-p75NTR (catalog AF1157; R&D Systems, Minneapolis, MN, US. catalog ab52987; Abcam), anti-TIM-1/KIM1/HAVCR (catalog AF1817; R&D Systems), Cy3-conjugated anti-α-smooth muscle actin (αSMA) (catalog C6198; Sigma-Aldrich), anti-CD11b (catalog 14-0112-82; eBioscience), anti-PDGFRβ (catalog 12-1402; eBioscience), and anti-CXCL13 (catalog MAB8012; R&D Systems). Staining was visualized using appropriate secondary antibodies. Lotus tetragonolobus lectin, FITC conjugate (catalog FL1321; Vector Laboratories, Newark, CA, USA) was also used for staining. Cell nuclei were counterstained with DAPI and mounted in mounting medium

(Fluoromount, catalog K024; Diagnostic BioSystems, Pleasanton, CA, USA). Immunofluorescence samples were analyzed using an OLYMPUS FV1000-D confocal microscope and Zeiss LSM900 confocal microscope.

Immunohistochemistry

For immunohistochemistry of mouse and human kidneys, formaldehyde-fixed and paraffin-embedded sections were deparaffinized and rehydrated, as described above, for immunofluorescence. After rehydration, endogenous peroxidase was blocked with 3% H2O2, and antigen retrieval was performed as described above for immunofluorescence. The sections were stained with the following primary antibodies: anti-8-Hydroxy-2'-deoxyguanosine [N45.1] (catalog ab48508; Abcam), anti-4-Hydroxynonenal (catalog ab46545; Abcam), anti-GPX4 (catalog ab125066; Abcam), anti-GCLM (catalog ZRB2011; Sigma-Aldrich), and anti-Cleaved caspase 3 (catalog 9664; CST). Antibody labeling was detected using a Histofine Simple Stain MAX-PO(R) (catalog 414341; NICHIREI BIOSCIENCES, Tokyo, Japan) and a Diaminobenzidine (DAB) Substrate Kit (catalog SK-4100; Vector Laboratories) for rabbit primary antibodies and a Histofine Simple Stain AP(M) (catalog 414241; NICHIREI BIOSCIENCES) and an ImmPACT Vector Red Substrate Kit (catalog SK-5105; Vector Laboratories) for mouse primary antibodies. Sections were counterstained using Mayer's Hematoxylin Solution (catalog 131-09665, FUJIFILM Wako Pure Chemical Corporation, Osaka, Japan) and mounted in malinol (catalog 20091; MUTO PURE CHEMICALS, Tokyo, Japan). For TUNEL staining, antigen retrieval was performed using 20 μg/mL Proteinase K (catalog 3115836001; Roche, Basel, Switzerland) at 37°C for 30 minutes. TUNEL staining was performed with the In Situ Cell Death Detection Kit, TMR Red (catalog 12156792910; Roche) according to the manufacturer's instructions.

RNAscope *in situ* hybridization

In situ hybridization was performed using RNAscope Multiplex Fluorescent Assay V2 (catalog 323100; Advanced Cell Diagnostics [ACD], Newark, CA, USA) on mouse kidney samples fixed with 4% paraformaldehyde for 24 h, OCT-embedded, cryosectioned at 10-μm thickness, or fixed with 10% neutralized formaldehyde for 24 h, paraffin-embedded, and sectioned at 4.0-μm thickness, according to the manufacturer's instructions. The following RNAscope Target Probes were used: Mm-Slc7a11 (catalog 42251; ACD) and Mm-Itgax-C2 (catalog 311501-C2; ACD).

Measurement of the size of tertiary lymphoid structures

The sizes of tertiary lymphoid structures in the kidney were examined in PAS-stained sections of injured kidneys as previously described[6]. Briefly, the size of tertiary lymphoid structures was defined as the total cumulative size of the sizes of tertiary lymphoid structures in the renal cortex of the sample. Images that included tertiary lymphoid structures were taken at the same size and resolution, and its size was measured by an experienced renal pathologist using the Adobe Photoshop software (Adobe Inc., San José, CA, USA) .

Real-time RT-PCR analysis

RNA extraction and real-time RT-PCR were performed as described previously[6]. The primer sequences are listed in the Supplementary Table S1. The expression levels were normalized to those of mouse *Gapdh*.

Imaging mass spectrometry

Matrix-assisted laser desorption/ionization-time of flight imaging Mass spectrometry (MALDI-IMS) of murine kidneys was performed as previously reported.[7,8] Briefly, freshly frozen tissues were sectioned at a thickness of 8.0 μm using a cryostat (CM 3050, Leica) . Frozen sections were thawed and mounted onto indium-tin-oxide (ITO) -coated glass slides (Bruker Daltonics, Billerica, MA, USA) , followed by spray-coating with 9-aminoacridine as the matrix (10 mg/mL, dissolved

in 80% ethanol). MALDI imaging was performed using a MALDI-TOF mass spectrometer (UltraFlextreme, Bruker Daltonics) equipped with an Nd:YAG laser and an Orbitrap mass spectrometer (Q-Exactive Focus, Thermo Fisher Scientific, Waltham, MA, USA) combined with atmospheric pressure matrix-assisted desorption/ionization (AP-SMALDI10, TransMIT). Data were acquired in the negative mode with raster scanning at a pitch distance of 50 μm. Image reconstruction was performed using the FlexImaging 4.1 software (Bruker Daltonics) and Image Quest 1.1 (Thermo Fisher Scientific). Amino acids were imaged by MALDI-IMS using an on-tissue chemical derivatization method as previously described.[9]

Desorption Electrospray Ionization-Multiple-Reaction-Monitoring Mass Spectrometry (DESI-MRM) data acquisition was performed by integrating a Waters DESI-XS ionization source with a Waters Xevo TQ Absolute (TqA) mass spectrometer. Methanol/water (98:2 v/v) was delivered from the Waters nanoAcquity Binary Solvent Manager through an M-Class Symmetry C18 column to the DESI sprayer at a flow rate of 2 μL/min. High voltage applied to the sprayer generated an electrospray that was pneumatically directed at a 75° angle toward the tissue section using a nitrogen gas stream. For glutathione detection, MRM parameters (m/z 307.76>178.81) were set in positive ion detection mode. The DESI spray voltage, Heater Transfer Line (HTL) temperature, and nitrogen gas flow were set to 0.8 kV, 150 °C, and 0.15 MPa, respectively, to ensure optimal performance in negative ionization mode. These optimized settings were saved in a Waters .ipr file. Prior to biological sample analysis, the motion and precise alignment of the x,y stage in the optimized DESI-MRM setup was verified using black ink (m/z 666.06) as a test analyte. The DESI-MRM experiment configuration was completed using HDImaging v1.7 and the DESI Method Editor (Waters).

Metabolome analysis

Comprehensive metabolome analysis of murine kidneys was performed as previously reported.[10] Briefly, frozen kidney tissues were dissolved in 500 μL of methanol containing internal standard

(IS) using a homogenizer (Finger Masher, AM79330, Sarstedt, Tokyo, Japan). Equal volumes of ultrapure water and 0.4× volume of chloroform were then added and mixed. The mixture was then centrifuged at 15,000 × *g* for 15 min at 4°C. The resulting supernatant was filtered through an ultrafilter tube (Ultrafree-M C, UFC3 LCC NB; Human Metabolome Technologies, Tsuruoka, Japan). The filtrate was then concentrated by nitrogen flow-assisted evaporation on a heating block (DTU-28N, TAITEC, Koshigaya City, Japan). The concentrated filtrate was resuspended in 50 µL of ultrapure water for subsequent liquid chromatography-tandem mass spectrometry (LC-MS/MS) and ion chromatography-high resolution (IC-HR)-MS analytical procedures.

The metabolome analysis of anionic metabolites was performed using an Orbitrap mass spectrometer (Q-Exactive Focus, Thermo Fisher Scientific) connected to a high-performance IC system (ICS-5000+, Thermo Fisher Scientific). The ion chromatography system was equipped with an anion electrolytic suppressor (Dionex AERS 500; Thermo Fisher Scientific) to convert the potassium hydroxide gradient to pure water before entering the mass spectrometer. Separation was performed using a Dionex IonPac AS11 -HC-4 µm IC column (Thermo Scientific) with an IC flow rate of 0.25 mL/min, and the post column was supplemented with a methanol make-up flow at 0.18 mL/min for IC separation. The concentration gradient conditions for potassium hydroxide were as follows: 1 mM to 100 mM (0-40 min), 100 mM (40-50 min), and 1 mM (50.1-60 min); column temperature was 30°C. The Orbitrap mass spectrometer was operated in ESI negative mode for all detections. A full mass scan (*m*/*z* 70-900) was performed at a resolution of 70,000. The automatic gain control target was set to 3×10^6 ions with a maximum ion implantation time of 100 ms. Ionization parameters of the ion source were as follows: spray voltage 3 kV, transfer temperature 320°C, S-Lens level 50, heater temperature 300°C, sheath gas 36, and aux gas 10.

LC-MS/MS was used for metabolomic analysis of cationic metabolites. A triple quadrupole mass spectrometer (LCMS-8060, SHIMADZU) equipped with a direct-going electrospray (ESI) ion source was used in the positive and negative ESI and multiple reaction monitoring modes.

Samples were prepared on a Discovery HS F5-3 column (2.1 ID × 150 mm L, 3-μm particle, Sigma-Aldrich) using a step gradient of mobile phase A (0.1% formic acid) and mobile phase B (0.1% acetonitrile). A step gradient of mobile phase A:mobile phase B was performed at a ratio of 100:0 (0-5 min), 75:25 (5-11 min), 65:35 (11-15 min), 5:95 (15-20 min), 100:0 (20-25 min), flow rate of 0.25 mL/min and column temperature of 40°C.

Oxidized lipid analysis

Lipids and oxidized lipids were extracted by adding 100 μL of a 1:1 (v/v) solution of 1-butanol and methanol, containing 100 μM butylated hydroxytoluene (BHT) and 100 μM ethylenediaminetetraacetic acid (EDTA), to the cell pellets. The mixture was vortexed for 10 s, sonicated for 15 min in an ice-cooled sonic bath, and then centrifuged at 16,000 × g for 10 minutes at 20 °C. The resultant supernatant was transferred into a 0.2 mL glass insert equipped with a Teflon-lined cap for subsequent LC ESI-MS analysis.

The analysis of (oxidized) lipids utilized a Q-Exactive Focus mass spectrometer (Thermo Fisher Scientific) linked to an Ultimate 3000 HPLC system (Thermo Fisher Scientific). Chromatographic separation was achieved on a Thermo Scientific Accucore C18 column (2.1 × 150 mm, 2.6 μm). Mobile phase A comprised 10 mM ammonium formate in 50% acetonitrile with 0.1% formic acid, while mobile phase B consisted of 2 mM ammonium formate in a blend of acetonitrile, isopropyl alcohol, and water (10:88:2, v/v/v) with 0.02% formic acid. The applied step gradient proceeded as follows: from 65:35 at onset to 40:60 over 0 to 4 min, shifting to 15:85 from 4 to 12 minutes, then to 0:100 from 12 to 21 minutes, maintained at 0:100 from 21 to 24 min, reverted to 65:35 over 24 to 24.1 min, and finally to 100:0 from 24.1 to 28 min, with a flow rate of 0.4 mL/min and a column temperature maintained at 35°C.

The Q-Exactive Focus mass spectrometer was operated in both ESI positive and negative ion modes. A full mass scan (m/z 250–1100) was followed by three rapid data-dependent MS/MS scans with resolutions of 70,000 and 17,500. The instrument settings included an automatic gain

control target of 1 × 106 ions and a maximum ion injection time of 100 ms. Ion source adjustments were set with a spray voltage of 3 kV, a transfer tube temperature of 285°C, an S-Lens level of 45, a heater temperature of 370°C, a sheath gas flow rate of 60, and an auxiliary gas flow rate of 20. Data analysis was conducted using Qual Browser (Thermo Fisher Scientific) specifically for the assessment of oxidized lipids.

Urine metabolome analysis

Frozen urine (50 μL) was mixed with 500 μL of methanol containing IS and a standard (2-morpholinoethanesulfonic acid), followed by the addition of equal volumes of ultrapure water and 0.4 volume of chloroform (LC/MS grade, FUJIFILM Wako Pure Chemical Corporation). The resulting suspension was centrifuged at 2,800 × *g* for 15 min at 4°C. After centrifugation, the aqueous phase was ultrafiltered in ultrafiltration tubes (Ultrafree MC-PLHCC; Human Metabolome Technologies). The filtrate was then concentrated by nitrogen flow-assisted evaporation on a heating block (DTU-28N, TAITEC). The concentrated filtrate was dissolved in 50 μL of ultrapure water and analyzed using IC-HR-MS and LC-MS/MS. Urinary metabolite levels were calculated as relative values and were corrected for urinary creatinine concentrations. Data were analyzed using MetaboAnalyst 5.0 software (https://www.metaboanalyst.ca).

Cell Culture

DC2.4 cells, mouse dendritic cells, were purchased from Sigma-Aldrich (catalog SCC142) and cultured with RPMI-1640 (catalog R0883; Sigma-Aldrich) supplemented with 10% FBS (catalog CCP-F BS-BR-500; Cosmo Bio, Tokyo, Japan), 1X L-Glutamine (catalog TMS-002-C; Sigma-Aldrich), 1X non-essential amino acids (catalog 06344-56; Nacalai Tesque, Kyoto, Japan), 1X HEPES Buffer Solution (catalog 17557-94; Nacalai Tesque), 100 μg/ml penicillin-streptomycin (catalog 26253-84; Nacalai Tesque), and 0.0054X β-Mercaptoethanol (catalog 133-14571; FUJIFILM Wako Pure Chemical Corporation), according to the manufacturer's instructions.

C3H10T1/2 cells, mouse embryonic fibroblasts, were purchased from the Health Science Research Resources Bank (HSRRB, Osaka, Japan) and cultured with DMEM low glucose (catalog 08490; Nacalai Tesque) supplemented with 10% FBS and 100 μg/ml penicillin-streptomycin. Cells were incubated at 37°C in a 5% CO_2 incubator.

Knockdown of Slc7a11 in DCs and fibroblasts by siRNA

Slc7a11 knockdown in DC2.4 cells and C3H10T1/2 cells was performed as previously described.[11] [12] Briefly, DC2.4 cells and C3H10T1/2 cells were seeded at a density of 4×10^4 cells/well in a 12-well plate using the complete media described earlier. After 48 hours, cells were transfected with ON-TARGETplus Mouse Slc7a11 SMARTpool siRNA (catalog L-047420-01-0005; Horizon Discovery, Cambridge, UK) or a non-targeting control siRNA (catalog D-001810-10-05; Horizon Discovery) using the Lipofectamine 3000 Transfection Kit (catalog L3000-015; Thermo Fisher Scientific), according to the manufacturer's instructions. After siRNA treatment, the culture medium of DC2.4 cells and C3H10T1/2 cells was replaced with fresh medium containing 8 ng/mL recombinant murine TNF-α (catalog 315-01A; PeproTech, Cranbury, NJ, USA) and 10 ng/mL IFN-γ (catalog 315-05; PeproTech) to induce xCT expression. Cells were harvested and analyzed 48 hours post-transfection.

T Cell Isolation from murine spleens by magnetic-activated cell sorting

T cells were isolated from murine spleen using magnetic-activated cell sorting (MACS) by negative selection according to the manufacturer's instructions, with the Pan T Cell Isolation Kit II, mouse (catalog 130-095-130; Miltenyi Biotec, Bergisch Gladbach, Germany). Spleens were harvested from mice and single-cell suspensions were prepared by gently mashing the tissue through a 40 μm cell strainer using the plunger of a syringe. Erythrocytes were lysed using RBC lysis buffer (catalog 130-094-183; Miltenyi Biotec) for 5 minutes at room temperature, followed by washing with MACS buffer (PBS containing 0.5% bovine serum albumin and 2 mM EDTA).

The resulting splenocytes were counted and resuspended in MACS buffer at a concentration of 10^8 cells per 400 μL. Cells were then incubated with a biotin-antibody cocktail (50 μL per 10^8 cells) for 5 minutes at 4°C to label non-T cells. Subsequently, anti-biotin microbeads (100 μL per 10^8 cells) were added and incubated for an additional 10 minutes at 4°C. The cell suspension was then passed through an LS column placed in a MACS separator. The flow-through containing the unlabeled T cells was collected. The purity of isolated T cells was confirmed by flow cytometry analysis using anti-CD3 antibody, consistently yielding >98% purity. The entire isolation procedure was completed within 4 hours to minimize cellular stress and maintain T cell functionality.

Co-culture Experiments

Co-culture experiment of T cells with DC2.4 cells and C3H10T1/2 cells were performed as previously described.[13] 48 hours post-siRNA treatment, the culture media of DC2.4 cells and C3H10T1/2 cells were removed and replaced with co-culture medium consisting of RPMI-1640 supplemented with 10% FBS, 1X L-Glutamine, 1X non-essential amino acids, 1X HEPES buffer solution, and 100 μg/mL penicillin-streptomycin, without β-Mercaptoethanol. T cells were then added to each well at a 1:4 ratio of DCs or fibroblasts to T cells and co-cultured for 24 hours at 37°C in a 5% CO_2 incubator. To maintain the viability of T cells, 1 μg/mL anti-CD3 antibody (catalog 16-0032-82; eBioscience) and 2 μg/mL anti-CD28 antibody (catalog 16-0281-85; eBioscience) were added to the co-culture medium. 24 hours after starting the co-culture, intracellular glutathione levels of T cells were measured by fluorescence-activated cell sorting (FACS) using the glutathione detection reagent ThiolTracker Violet (catalog T10095; Invitrogen, Waltham, MA, USA) . For inducing oxidative stress, we treated cells by 200 μM tert-Butyl Hydroperoxide (TBHP, catalog 180342500; Thermo Fisher Scientific) at 37°C for 1 hour and analyzed intracellular glutathione levels. For sulfasalazine treatment, dendritic cells or fibroblasts were pre-treated with 500 μM sulfasalazine or vehicle (DMSO) for 24 hours, based on a previous

study,[13] before initiating co-culture with T cells. Intracellular glutathione levels were then analyzed after 24 hours using ThiolTracker Violet as described above.

Mononuclear cell isolation from the kidney, spleen, and peripheral blood

Single-cell suspensions of the kidneys, spleen, and peripheral blood were obtained in the previously described method.[4] Briefly, after the collection of 100 μL of blood, mice were perfused with Hank's Balanced Salt Solution (HBSS, catalog 17461-05; Nacalai Tesque) and then the kidneys and spleens were isolated. The kidneys were minced finely and homogenated in 5 ml of FACS buffer (HBSS with 2% FCS and 1% EDTA). The homogenate was centrifuged at 500g for 7 minutes under 4°C and resuspended in 5ml of collagenase I solution (1 mg/mL collagenase in HBSS) for 15 minutes at 37°C, with gentle stirring. Digested kidneys were then centrifuged at 400g for 5 minutes. The resulting pellets were resuspended in 10 mL of FACS buffer and incubated on ice for 15 minutes. Suspended solution was filtered through a 70-μm cell strainer. The suspension is centrifuged at 400g for 5 minutes under 4°C and resuspended in 1mL of RBC lysis buffer for 1 minutes. 4 mL of FACS buffer was added and centrifuged at 400g for 5 minutes. A single-cell suspension of the kidney digestion was obtained by resuspending the pellet in 1 mL of FACS buffer and filtering through a 70-μm cell strainer.

The spleens were mechanically minced firmly using the plunger of a syringe in 4 mL of FACS buffer and filtered through a 40-μm cell strainer. The suspension was centrifuged at 400g for 5 minutes under 4°C and resuspended in 1 mL of FACS buffer. 4mL of RBC lysis buffer was added and incubated for 2 minutes under room temperature. The suspension was centrifuged at 400g for 5 minutes under 4°C and washed twice. A single-cell suspension of splenocytes was generated by resuspending the pellet in 1 mL of FACS buffer.

For peripheral blood, 1 mL of FACS buffer was added to 100 μL of peripheral blood with 10 U of heparin sodium (catalog 224122458; Mochida, Japan) and centrifuged at 400g for 5 minutes under 4°C. 1 mL of RBC lysis buffer was added and incubated for 10 minutes under room

temperature. The solution was centrifuged at 400g for 5 minutes under 4°C. A single-cell suspension of blood cells was obtained by resuspending the pellet in 1 mL of FACS buffer.

Flow cytometric analysis

Single-cell suspensions were blocked with FcR Blocking Reagent (Miltenyi Biotec) at 4°C for 10 minutes, followed by staining with a cocktail of conjugated antibodies against the following proteins: mouse CD45 (clone 30-F11, Pacific Blue conjugate, catalog 103112, and APC-Cy7 conjugate, catalog 103115; BioLegend, San Diego, CA, USA) , CD3ε (clone 145-2C11, FITC conjugate, catalog 100306; BioLegend) , CD19 (clone 1D3, PE conjugate, catalog 152408; BioLegend) , CD11b (clone M1/70, APC conjugate, catalog 101211; BioLegend) , and CD11c (clone N418, APC-Cy7 conjugate, catalog 117323; BioLegend) . Staining was performed at 4°C for 30 minutes. For oxidative stress measurement, cell suspensions were first incubated with 5 μM CellRox Green Reagent (catalog C10444; Invitrogen) at 37°C for 30 minutes, followed by antibody staining. For intracellular glutathione level measurement in co-cultured T cells using ThiolTracker Violet, cells were first stained with the antibody cocktail, followed by incubation with 10 μM ThiolTracker Violet at 37°C for 30 minutes. T cells were isolated by excluding CD11b (+) CD11c (+) cells in co-culture with DCs, or by including CD45 (+) cells in co-culture with fibroblasts. Flow cytometric analyses were conducted using a FACSAria Fusion (BD Biosciences) and analyzed with FlowJo software (Becton Dickinson Co.) .

Reanalysis on single-nucleus RNA-sequencing dataset of aged injured kidneys with tertiary lymphoid structures

We reused the single-nucleus RNA-sequencing Seurat object of aged injured kidneys with tertiary lymphoid structures 30 days after a 45-minute IRI that we previously reported.[14] We showed Uniform Manifold Approximation and Projection (UMAP) plots and gene expression patterns in each cluster.

Statistics

Results are presented as the mean ± SE. Statistical analysis was performed with non-parametric tests, using Mann-Whitney U test (Figures 1J, 4C, 5D, 6A, 6B, 6G, Supplemental Figure 3B, 8C, 9C, 10B, 10E, 11, 14), multiple Mann-Whitney U test (Figure 5B), Steel test (Figures 4E, 4F, 4H, 6C, Supplemental Figure 4B, 8E, 9E, 9F, 13), non-parametric trend test (Figure 3A, 3E, 3I, 3M, Supplemental Figure 6B), 2-way ANOVA after aligned rank transform[15] (Figure 5E), nonparametric multiple comparison with Dunnett-type contrasts using the nparcomp package in R[16] (Figure 1G), Pearson's chi-square test (Table 1), Wilcoxon rank-sum test (Table 1, Supplemental Figure 16, Supplemental Table 1), and multivariate logistic regression analysis (Figure 6H and Table 2) as indicated in the figure legends, with significance set at $P < 0.05$. Statistical analyses were performed using JMP version 11 (SAS Institute Inc., Cary, NC, USA), GraphPad Prism for Mac (version 10; GraphPad Software, La Jolla, CA, USA), and R v4.4.0.

All patients followed by steroid treatment (n=9)			
	Baseline	One year	p value
Serum creatinine (mg/dL)	1.31 (0.86-1.06)	1.27 (0.82-1.12)	0.840
eGFR (mL/min/1.73m^2)	57.6 (52.6-69.2)	56.8 (49.7-70.3)	0.820
UPCR (g/gCr)	1.71 (0.35-2.00)	0.62 (0.12-0.39)	0.004

Responders to steroid treatment (n=7)			
	Baseline	One year	p value
Serum creatinine (mg/dL)	1.44 (0.91-1.56)	1.38 (0.89-1.58)	0.625
eGFR (mL/min/1.73m^2)	49.7 (36.4-59.0)	49.6 (35.3-66.3)	>0.999
UPCR (g/gCr)	1.451(0.51-1.94)	0.27 (0.14-0.34)	0.016

Supplemental table 1: Changes in serum creatinine, eGFR, and urinary protein levels in IgA nephropathy patients at baseline and one year after renal biopsy.

Of the 18 patients with tertiary lymphoid structures in the kidney recruited in Figure 6, nine patients were treated by steroid therapy and had urine samples available at both the time of diagnosis and one year later. Seven patients showed a favorable response to steroid treatment, with more than a 50% reduction in urinary protein levels.

Data are median (25th-75th percentile) or percentage.

Data were analyzed by Wilcoxon rank-sum test.

eGFR: Estimate glomerular filtration rate. UPCR: Urinary protein to creatinine ratio.

Supplemental Table 2: Primer sequences used for real-time PCR

Gene	Sequence (5′-3′)	
	Forward	Reverse
Gapdh	ACGGCAAATTCAACGGCACAGTCA	TGGGGGCATCGGCAGAAGG
Slc7a11	CTTTTGTTCGAGTCTGGGTGGAAC	GTTCCAGGATGTAGCGTCCAAATG
Abcc1	ACAACCTGCGCTTCAAGATCAC	AAAGCCCTTTAGGTGAGCAAGC
Cxcl13	CGTGCCAAATGGTTACAAAGATT	GTGGCTTCAGGCAGCTCTTC
Ccl19	CCTGGGAACATCGTGAAAGC	TGGAGGTGCACAGAGCTGATA
Ifng	CTCATGGCTGTTTCTGGCTGTTAC	TTTCTTCCACATCTATGCCACTTG
Ngfr	AGAGAAACTGCACAGCGACAG	TAGAGGTTGCCATCACCCTTGAG
Cd4	AAGAACTGGTTCGGCATGACAC	TGCAAAGTTGAGTGGGAAGGAG
Cd19	CCTGGGCATCTTGCTAGTGATTG	AAGCATTCCACCGGAACATCTC
Itgax	TCTTGGTCTGAACAAGCACTGTG	AAGTTGAGGCGAAGAGTGATCG
Havcr1	TCTATGTTGGCATCTGCATCG	GAAGGCAACCACGCTTAGAGA
Col1a1	AGCCGCAAAGAGTCTACATGTC	TAGGCCATTGTGTATGCAGCTG
Gpx4	ATGCACGAATTCTCAGCCAAGG	TTGGTGACGATGCACACGAAAC
Acsl4	CGACAACATCTGTGCTTTTGCC	AGCTTCCATGGCGGGATTATTG

References for Supplemental Information

1. Yamauchi Y, Abe K, Mantani A, et al. A novel transgenic technique that allows specific marking of the neural crest cell lineage in mice. *Dev Biol.* 1999;212(1):191-203.
2. Luche H, Weber O, Nageswara Rao T, Blum C, Fehling HJ. Faithful activation of an extra-bright red fluorescent protein in "knock-in" Cre-reporter mice ideally suited for lineage tracing studies. *Eur J Immunol.* 2007;37(1):43-53.
3. Sato Y, Boor P, Fukuma S, et al. Developmental stages of tertiary lymphoid tissue reflect local injury and inflammation in mouse and human kidneys. *Kidney Int.* 2020;98(2):448-463.
4. Sato Y, Oguchi A, Fukushima Y, et al. CD153/CD30 signaling promotes age-dependent tertiary lymphoid tissue expansion and kidney injury. *J Clin Invest.* 2022;132(2).
5. Iguchi T, Takaori K, Mii A, et al. Glucocorticoid receptor expression in resident and hematopoietic cells in IgG4-related disease. *Mod Pathol.* 2018;31(6):890-899.
6. Sato Y, Mii A, Hamazaki Y, et al. Heterogeneous fibroblasts underlie age-dependent tertiary lymphoid tissues in the kidney. *JCI Insight.* 2016;1(11):e87680.
7. Kunisawa J, Sugiura Y, Wake T, et al. Mode of Bioenergetic Metabolism during B Cell Differentiation in the Intestine Determines the Distinct Requirement for Vitamin B1. *Cell Rep.* 2015;13(1):122-131.
8. Sugiura Y, Katsumata Y, Sano M, et al. Visualization of in vivo metabolic flows reveals accelerated utilization of glucose and lactate in penumbra of ischemic heart. *Sci Rep.* 2016;6:32361.
9. Toue S, Sugiura Y, Kubo A, et al. Microscopic imaging mass spectrometry assisted by on-tissue chemical derivatization for visualizing multiple amino acids in human colon cancer xenografts. *PROTEOMICS.* 2014;14(7-8):810-819.
10. Maeda R, Seki N, Uwamino Y, et al. Amino acid catabolite markers for early prognostication of pneumonia in patients with COVID-19. *Nat Commun.* 2023;14(1):8469.
11. Ruan Z, Takamatsu-Yukawa K, Wang Y, et al. Functional genome-wide short hairpin RNA library screening identifies key molecules for extracellular vesicle secretion from microglia. *Cell Rep.* 2022;39(6):110791.
12. Dong G, Adak S, Spyropoulos G, et al. Palmitoylation couples insulin hypersecretion with β cell failure in diabetes. *Cell Metab.* 2023;35(2):332-344.e337.
13. Yan Z, Garg SK, Kipnis J, Banerjee R. Extracellular redox modulation by regulatory T cells. *Nat Chem Biol.* 2009;5(10):721-723.
14. Yoshikawa T, Oguchi A, Toriu N, et al. Tertiary Lymphoid Tissues Are Microenvironments with Intensive Interactions between Immune Cells and Proinflammatory Parenchymal

Cells in Aged Kidneys. *J Am Soc Nephrol.* 2023.

15. Wobbrock JO, Findlater L, Gergle D, Higgins JJ. The aligned rank transform for nonparametric factorial analyses using only anova procedures. Proceedings of the SIGCHI Conference on Human Factors in Computing Systems; 2011; Vancouver, BC, Canada.

16. Konietschke F, Placzek M, Schaarschmidt F, Hothorn LA. nparcomp: An R Software Package for Nonparametric Multiple Comparisons and Simultaneous Confidence Intervals. *Journal of Statistical Software.* 2015;64(9):1 - 17.

Supplemental Figure 1

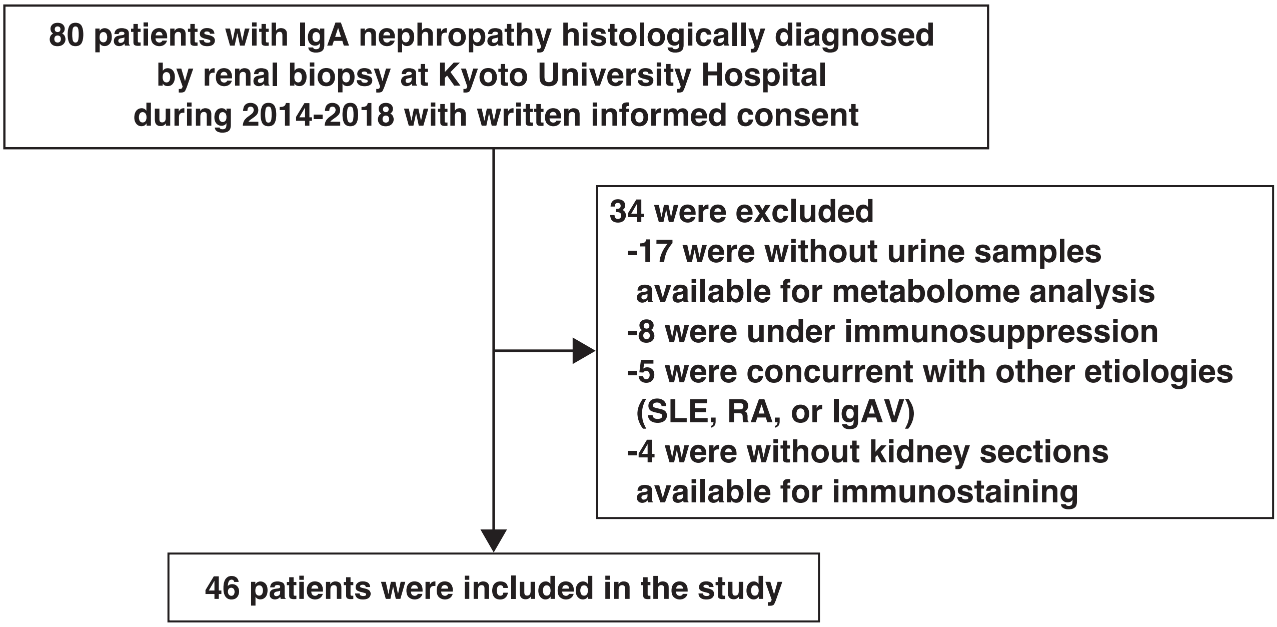

Supplemental Figure 1. Enrollment for histological evaluation of tertiary lymphoid structures in the kidney and metabolome analysis of urine samples.
80 patients were diagnosed as IgA nephropathy by renal biopsy at Kyoto University Hospital between 2014 and 2018 with written informed consent for the use of biosamples in the clinical study. 46 patients were finally included in the study to evaluate tertiary lymphoid structures in the kidney and analyze urine metabolite profiles.
LN: Lupus nephritis, RA: Rheumatoid arthritis, IgAV: IgA vasculitis.

Supplemental Figure 2

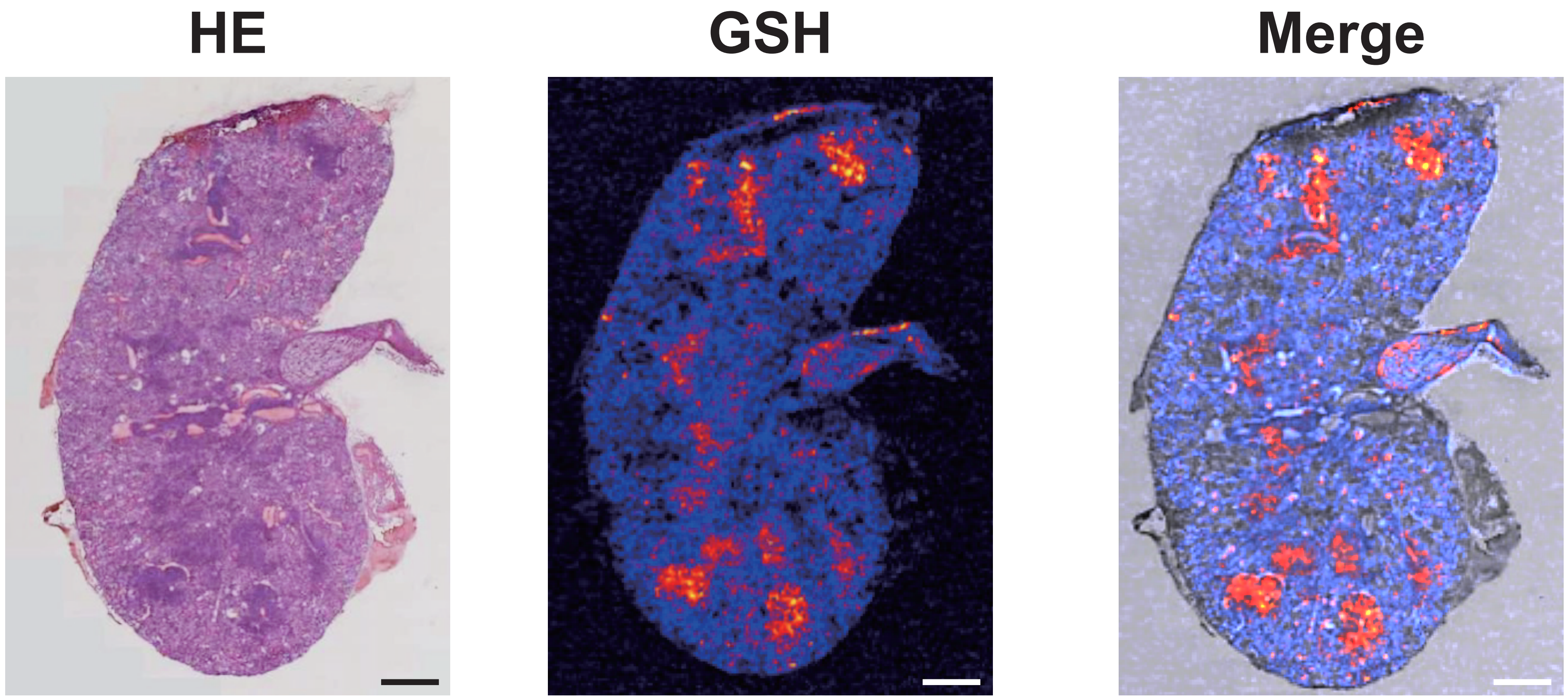

Supplemental Figure 2: DESI-imaging mass spectrometry of GSH in the kidney with tertiary lymphoid structures.

HE staining and Desorption Electrospray Ionization (DESI) – Imaging Mass Spectrometry of GSH in the kidneys 45 days after IRI. GSH accumulation was observed within tertiary lymphoid structures. Scale bars: 1000 μm.

Supplemental Figure 3

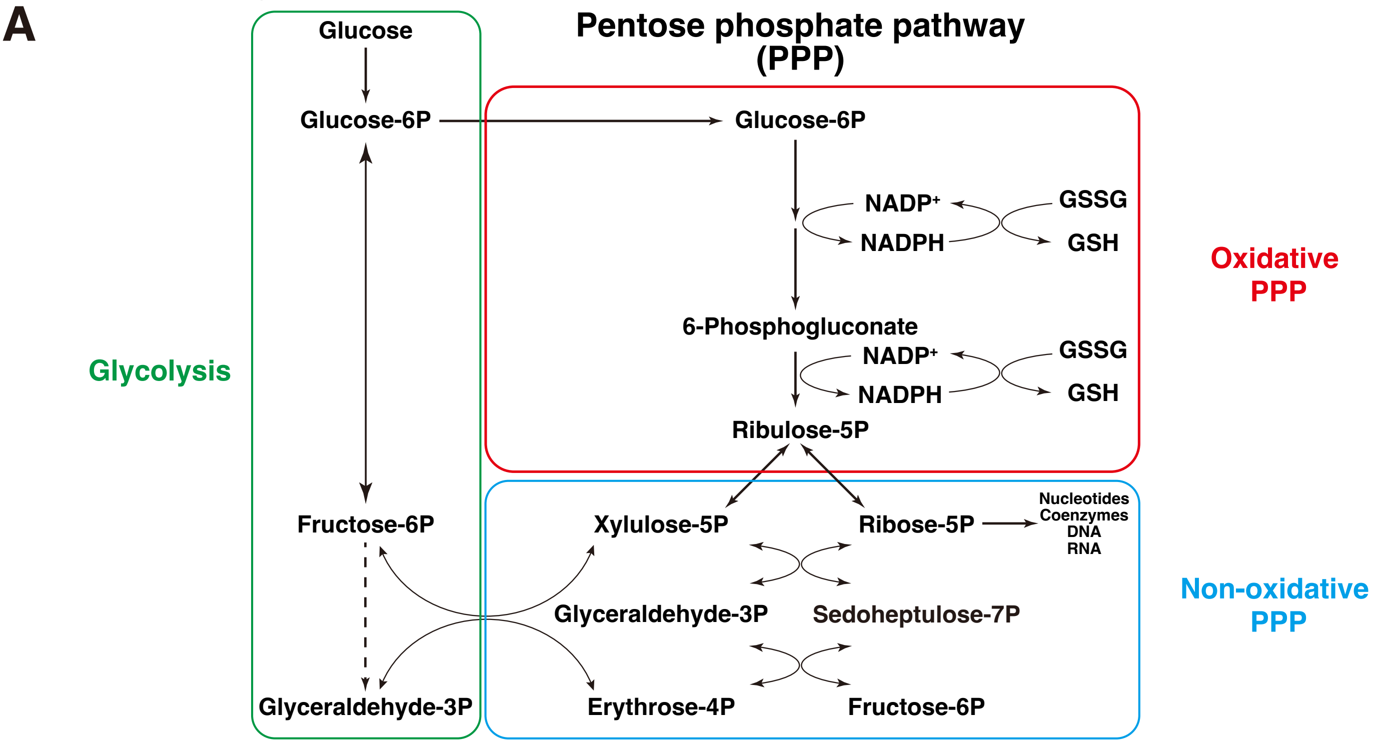

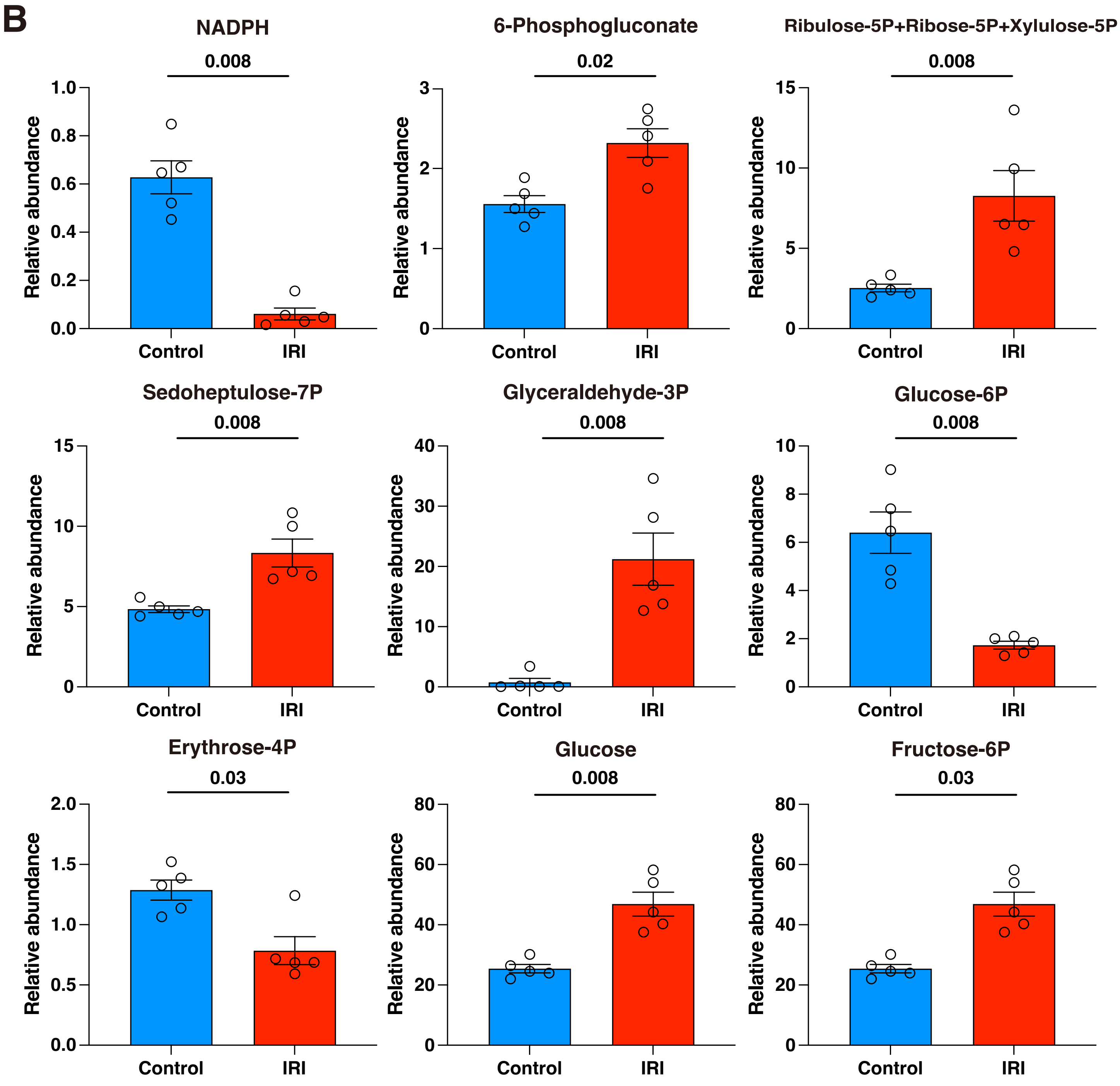

Supplemental Figure 3. Pentose phosphate pathway is upregulated in tertiary lymphoid structures in the kidney.

(A) Overview of the pentose phosphate pathway (PPP) with key metabolites. NADPH is generated via oxidative PPP and used for the conversion of GSSG to GSH. Ribose-5P, which is essential for the synthesis of nucleotides and cell proliferation, is produced via non-oxidative PPP.

(B) Metabolome analysis of key metabolites in PPP in the kidneys 45 days after sham surgery and IRI (n = 5 in each group).

Values are means ± SE. Data were analyzed by Mann-Whitney U test.

Supplemental Figure 4

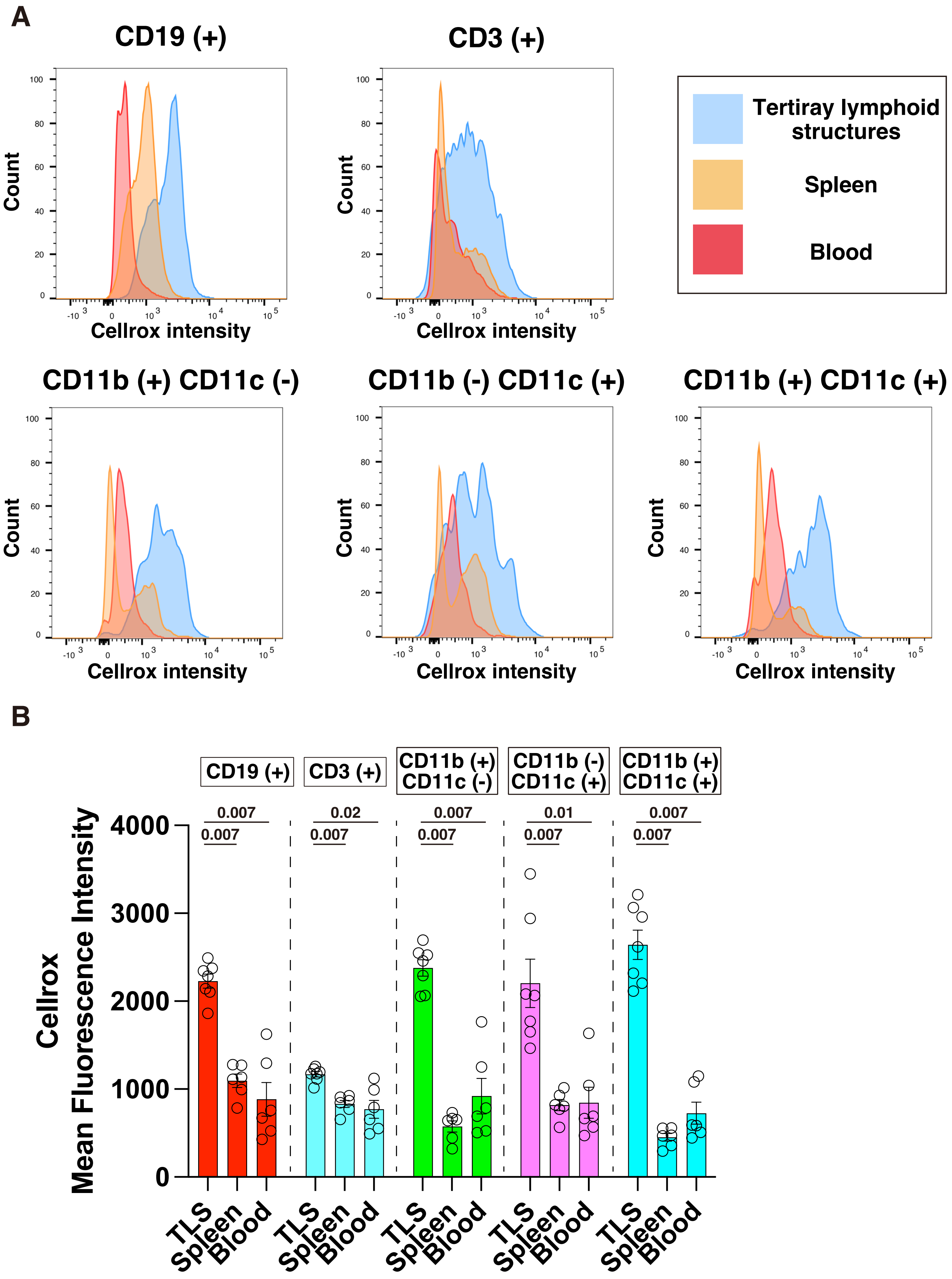

Supplemental Figure 4. Higher oxidative stress accumulates in immune cells within tertiary lymphoid structures compared with those within the spleen and peripheral blood.

(A) Representative histogram of Cellrox intensity in CD19 (+), CD3 (+), CD11b (+) CD11c (-), CD11b (-) CD11c (+), and CD11b (+) CD11c (+) cells derived from tertiary lymphoid structures (n = 7), spleen (n = 6), and peripheral blood (n = 6) of aged mice after 45 minute unilateral IRI.

(B) The quantitative mean fluorescence intensity (MFI) of Cellrox in each immune cell subpopulation derived from tertiary lymphoid structures, the spleen, and peripheral blood.

Cells are gated on CD45 (+) and analyzed accordingly.

Values are means ± SE. Data were analyzed by (B) Steel test with the group of tertiary lymphoid structures as control. TLS, tertiary lymphoid structures.

Supplemental Figure 5

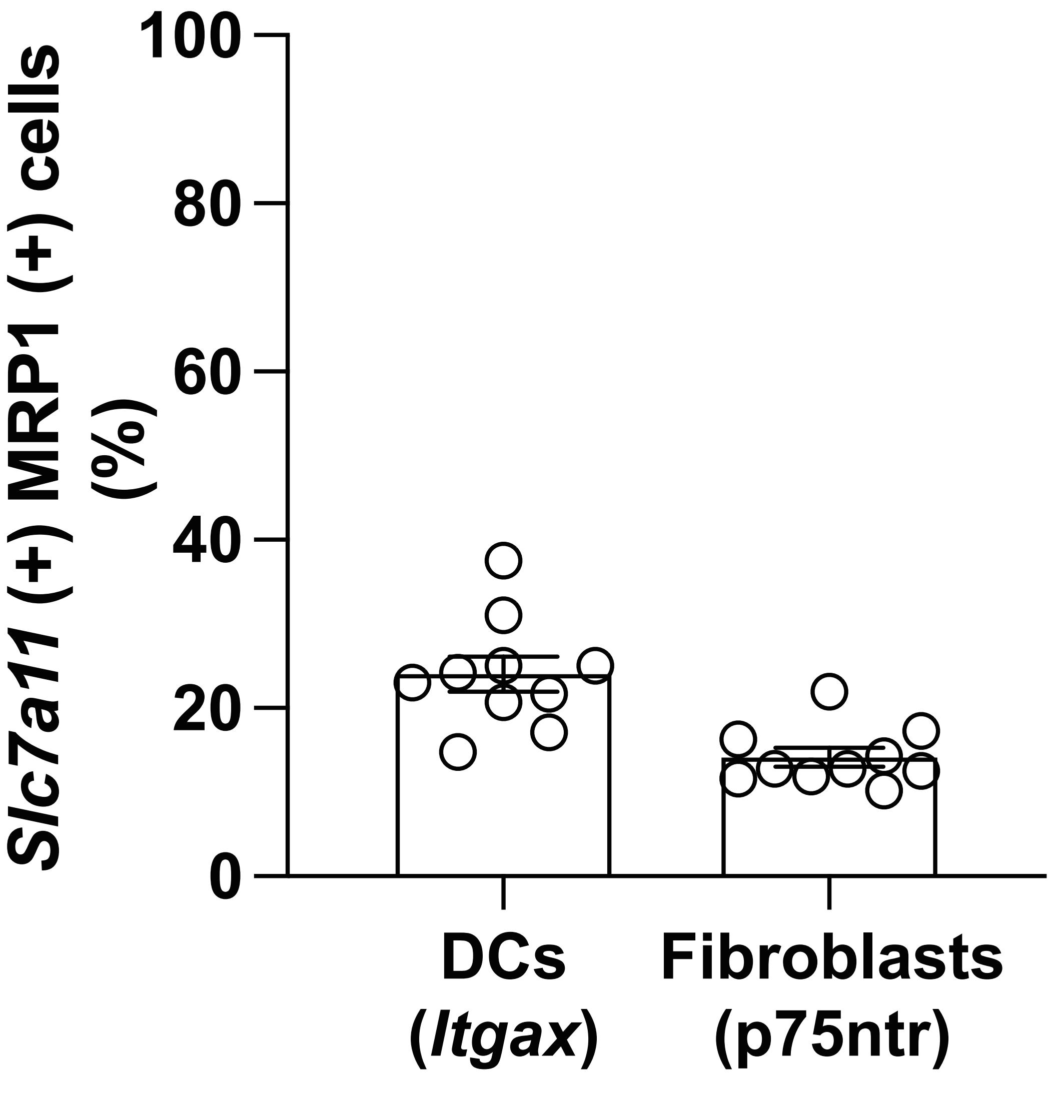

Supplemental Figure 5. The percentages of *Slc7a11* (+) MRP1 (+) dendritic cells and fibroblasts within TLSs.

The number of *Slc7a11* (+) MRP1 (+) *Itgax* (+) cells and *Slc7a11* (+) MRP1 (+) p75ntr (+) cells within TLSs were counted and the percentage of these cells per *Itgax* (+) or p75ntr (+) cells were calculated. Ten TLS lesions from five mice were randomly selected and used for analysis. Counting and calculation was performed by using ImageJ software.

Supplemental Figure 6

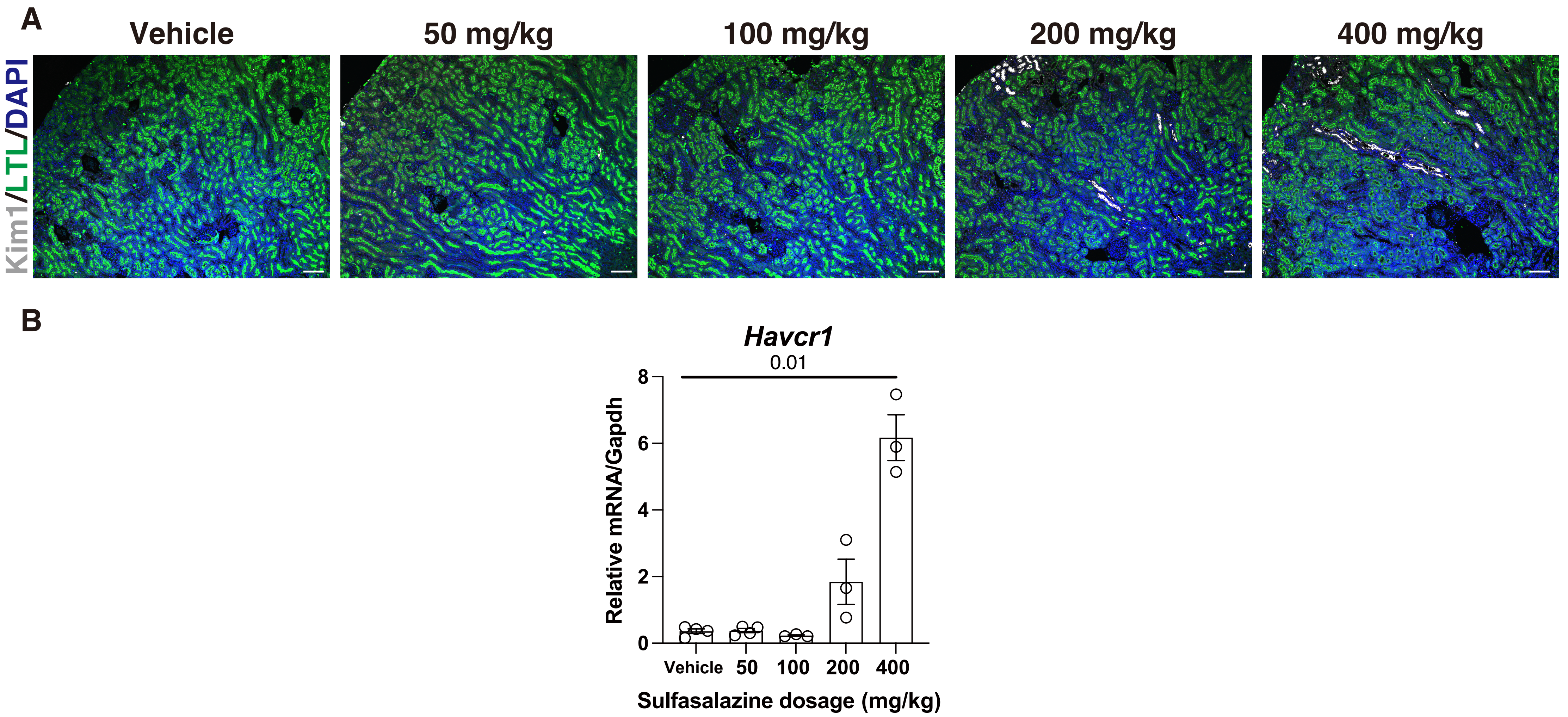

Supplemental Figure 6. Tubular injury in the kidney following different dosages of sulfasalazine-treatment.

(A) Immunofluorescence of Kim1 (white), LTL (green), and DAPI (blue) in the kidneys of mice treated daily with varying dosages of sulfasalazine for 16 days. The 400g/kg dosage corresponds to that used in the experiment described in Figure 4.

(B) *Havcr1* mRNA levels in the kidneys of mice treated with varying dosages of sulfasalazine (Vehicle: n = 4; 50mg/kg: n = 3; 100mg/kg: n = 3; 200mg/kg: n=3; and 400mg/kg: n = 3).

Values are means ± SE. Data were analyzed by non-parametric trend test.

Scale bars: 100 μm.

Supplemental Figure 7

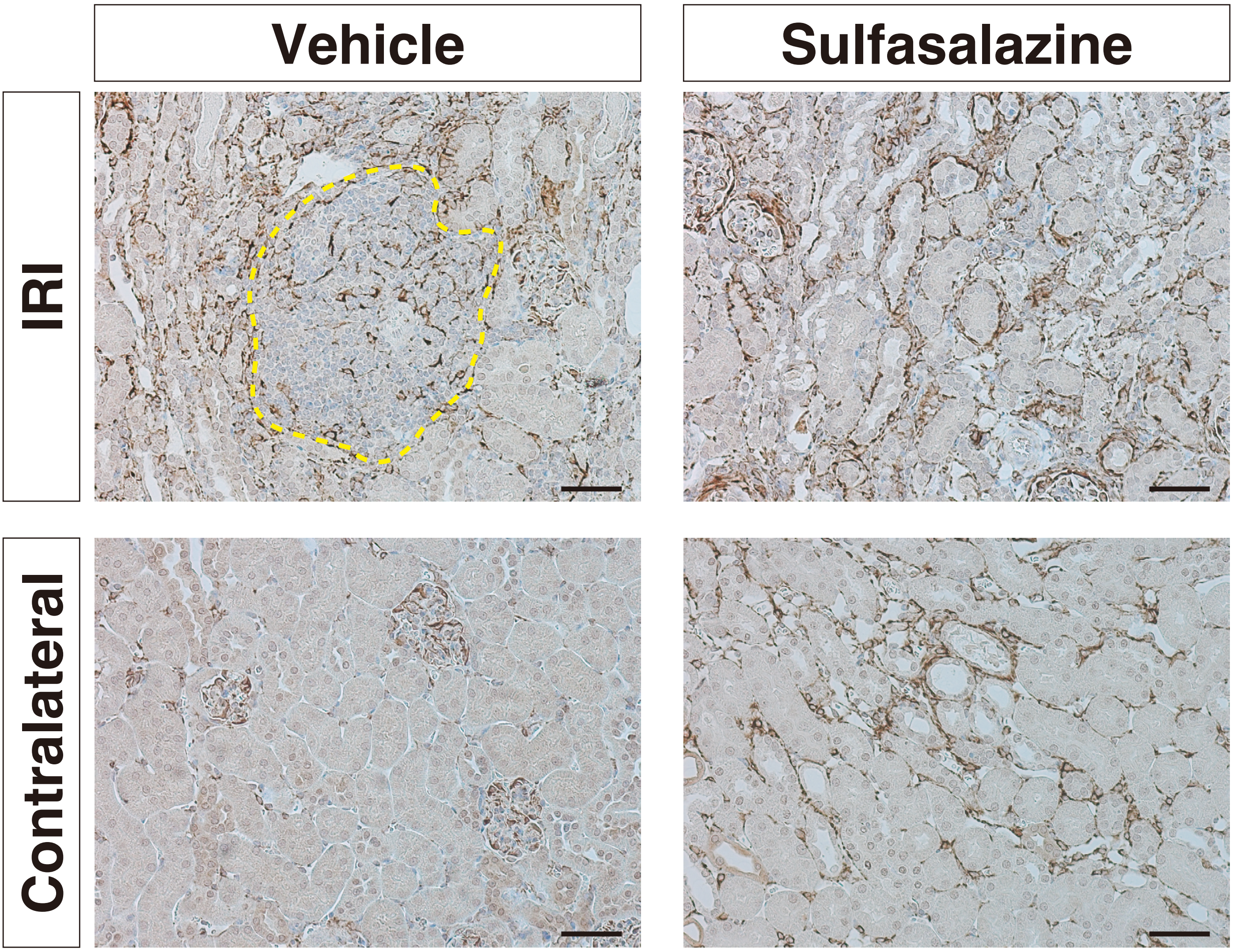

Supplemental Figure 7. Desmin expression in the kidney of mice treated with sulfasalazine or vehicle after IRI.

Desmin expression was analyzed in the kidneys at Day30 after IRI treated with sulfasalazine or vehicle. Both the IRI-affected kidneys and contralateral kidneys were examined. Desmin was detected by DAB staining (brown). Sections were counterstained with eosin. Tertiary lymphoid structures are encircled by dotted lines.

Scale bars: 50 μm.

Supplemental Figure 8

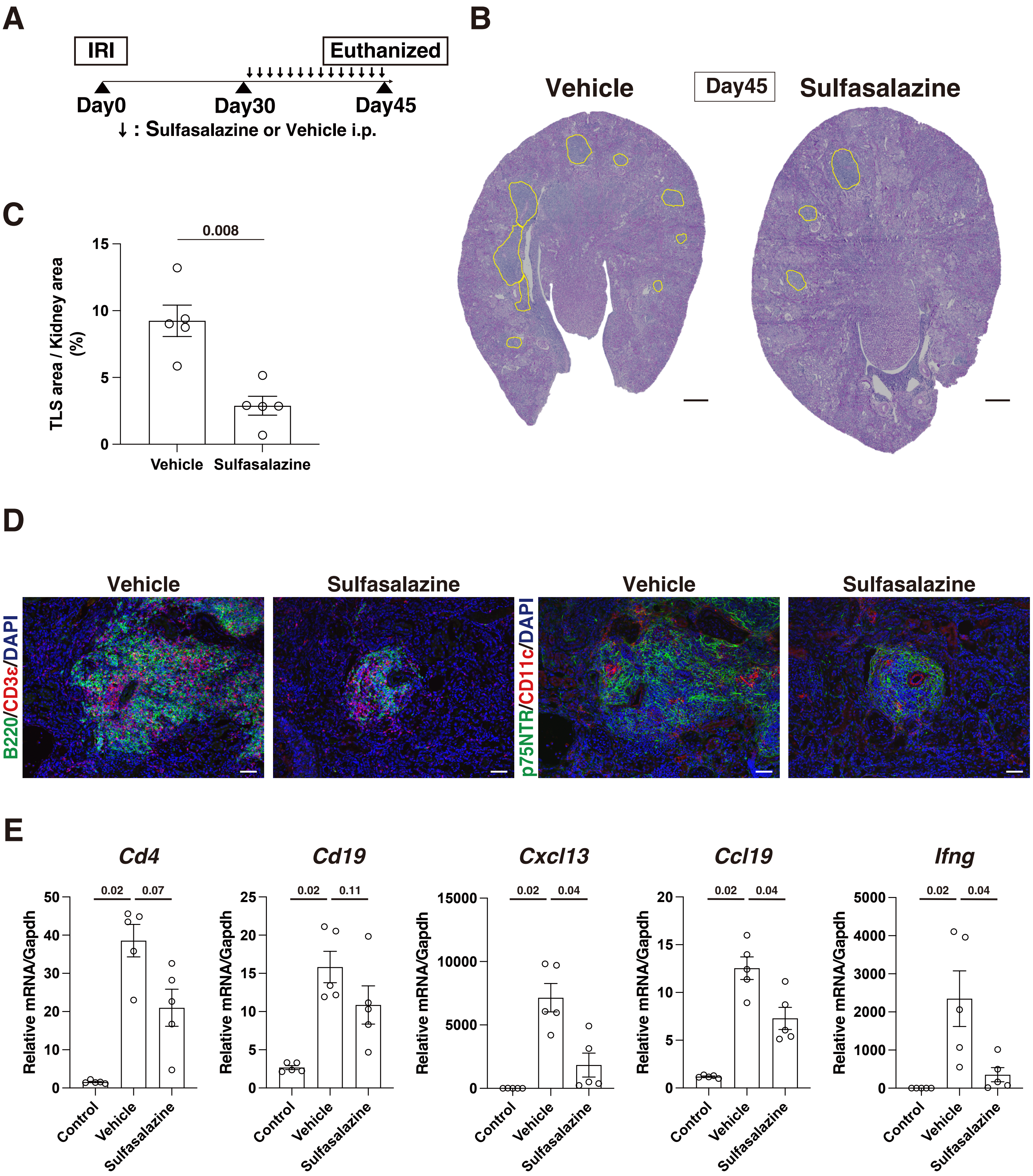

Supplemental Figure 8. Sulfasalazine treatment contributes to partial reversion of tertiary lymphoid structures in the kidney.

(A) Experimental protocol for (B-E).

(B) Representative images of the kidneys 45 days after IRI treated with sulfasalazine or vehicle from day 30 in Periodic acid–Schiff (PAS) staining. Tertiary lymphoid structures are encircled by yellow lines.

(C) Cumulative sizes of tertiary lymphoid structures per kidney cortex area (n = 5 in each group).

(D) Immunofluorescence of B220 (green), CD3□ (red), and DAPI (blue) and p75NTR (green), CD11c (red), and DAPI (blue) in the kidneys 45 days after IRI treated with sulfasalazine or vehicle from day 30.

(E) *Cd4*, *Cd19*, *Cxcl13*, *Ccl19*, and *Ifng* mRNA levels in the IRI kidneys (Control: kidneys 45 days after sham operation, n = 5; Vehicle: kidneys 45 days after IRI treated with vehicle from day30, n = 5; Sulfasalazine: the kidneys 45 days after IRI treated with sulfasalazine 400 mg/kg from day 30, n = 5).

Values are means ± SE. Data were analyzed by (C) Mann-Whitney U test and (E) Steel test with the vehicle-treated group as control.

Scale bars: (B) 300 μm; (D) 50 μm.

Supplemental Figure 9

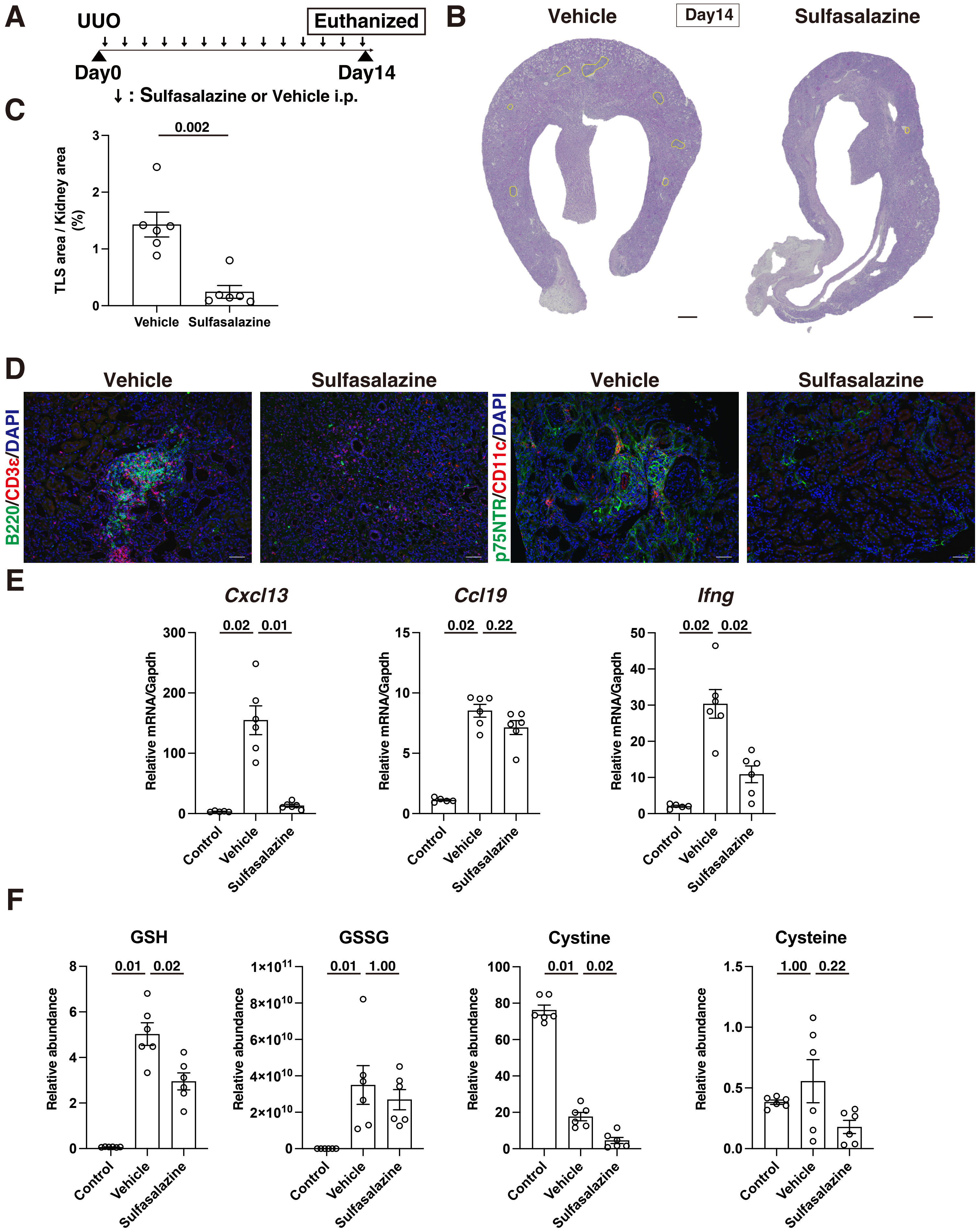

Supplemental Figure 9. Sulfasalazine treatment prevents the formation of tertiary lymphoid structures in unilateral ureteral obstruction model.

(A) Experimental protocol for (B-F).

(B) Representative images of the kidneys 14 days after unilateral ureteral obstruction (UUO) treated with sulfasalazine or vehicle in Periodic acid–Schiff (PAS) staining. Tertiary lymphoid structures are encircled by yellow lines.

(C) Cumulative sizes of tertiary lymphoid structures per kidney cortex area (n = 6 in each group).

(D) Immunofluorescence of B220 (green), CD3ε (red), and DAPI (blue) and p75NTR (green), CD11c (red), and DAPI (blue) in the kidneys 14 days after UUO treated with sulfasalazine or vehicle.

(E) *Cxcl13*, *Ccl19*, and *Ifng* mRNA levels in the kidneys after UUO (Control: normal kidneys, n = 5; Vehicle: kidneys 14 days after UUO treated with vehicle, n = 6; Sulfasalazine: the kidneys 14 days after UUO treated with sulfasalazine 400 mg/kg, n = 5).

(F) GSH, GSSG, cystine, and cysteine levels in the kidneys after UUO (n = 6 in each group).

Values are means ± SE. Data were analyzed by (C) Mann-Whitney U test and (E, F) Steel test with the vehicle-treated group as control.

UUO, Unilateral ureteral obstruction. GSH, Glutathione reduced form. GSSG, Glutathione oxidized form.

Scale bars: (B) 300 μm; (D) 50 μm.

Supplemental Figure 10

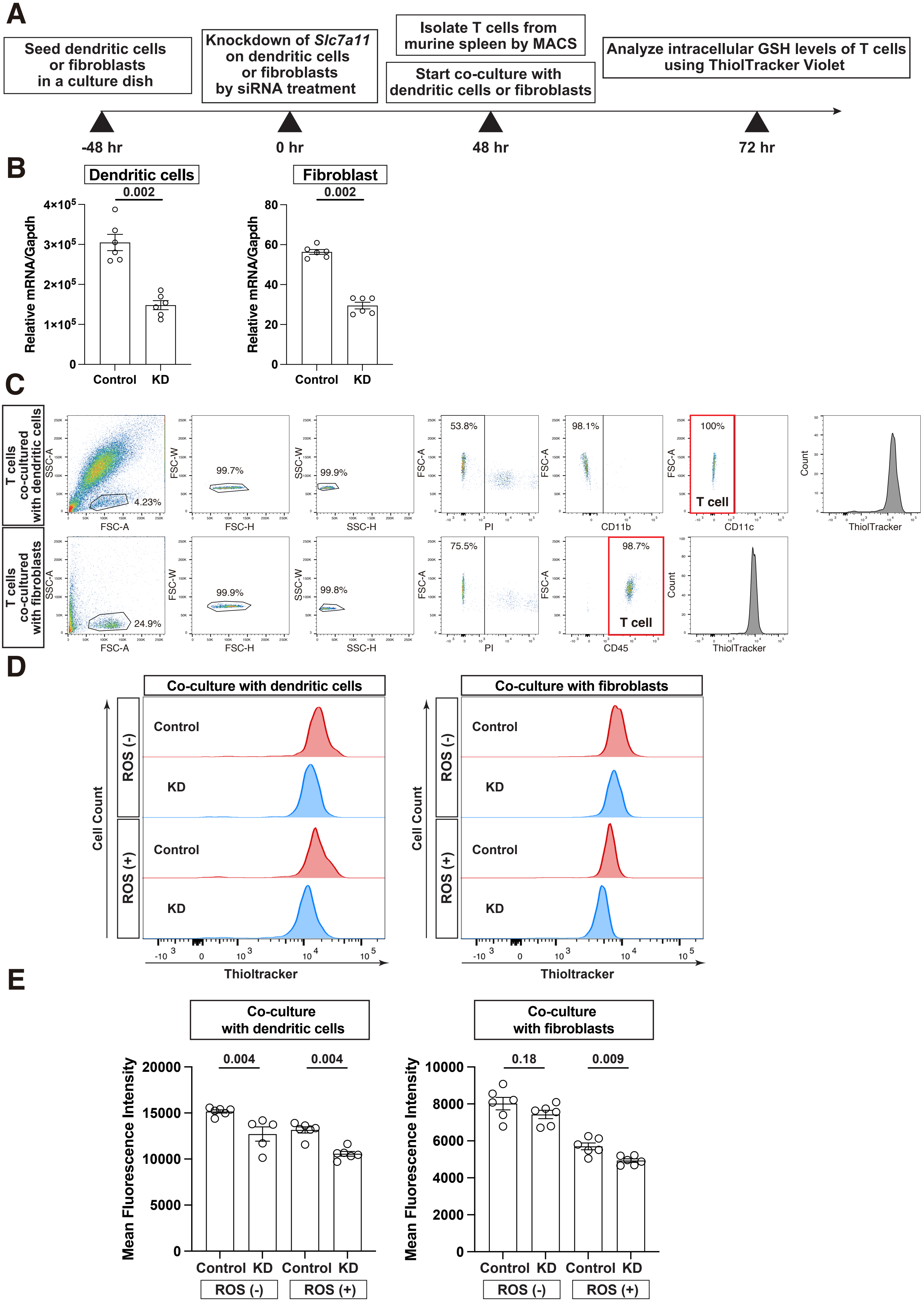

Supplemental Figure 10. Intracellular glutathione levels significantly decrease in T cells co-cultured with *Slc7a11*-knockdown dendritic cells or fibroblasts.

(A) Experimental protocol for (B-E).

(B) *Slc7a11* mRNA levels in dendritic cells and fibroblasts treated with *Slc7a11* siRNA or non-targeting probe (n = 6 in each group).

(C) Representative FACS plot for the identification of T cells co-cultured with dendritic cells or fibroblasts and the evaluation of ThiolTracker Violet (glutathione detection reagent) in T cells. T cells co-cultured with dendritic cells or fibroblast were identified by excluding CD11b (+) and CD11c (+) cells or including CD45 (+) cells, respectively.

(D) Representative histogram of mean fluorescence intensity (MFI) of ThiolTracker Violet in T cells co-cultured with dendritic cells or fibroblasts under normal condition or oxidative stress (after TBHP treatment).

(E) The quantitative MFI of ThiolTracker Violet in T cells co-cultured with dendritic cells or fibroblasts (n = 5 or 6 in each group).

Values are means ± SE. Data were analyzed by (B, E) Mann-Whitney U test.

KD, Knockdown; MACS, Magnetic activated cell sorting; FACS, Fluorescence activated cell sorting; ROS, redox oxidative stress; PI, Propidium iodide.

Supplemental Figure 11

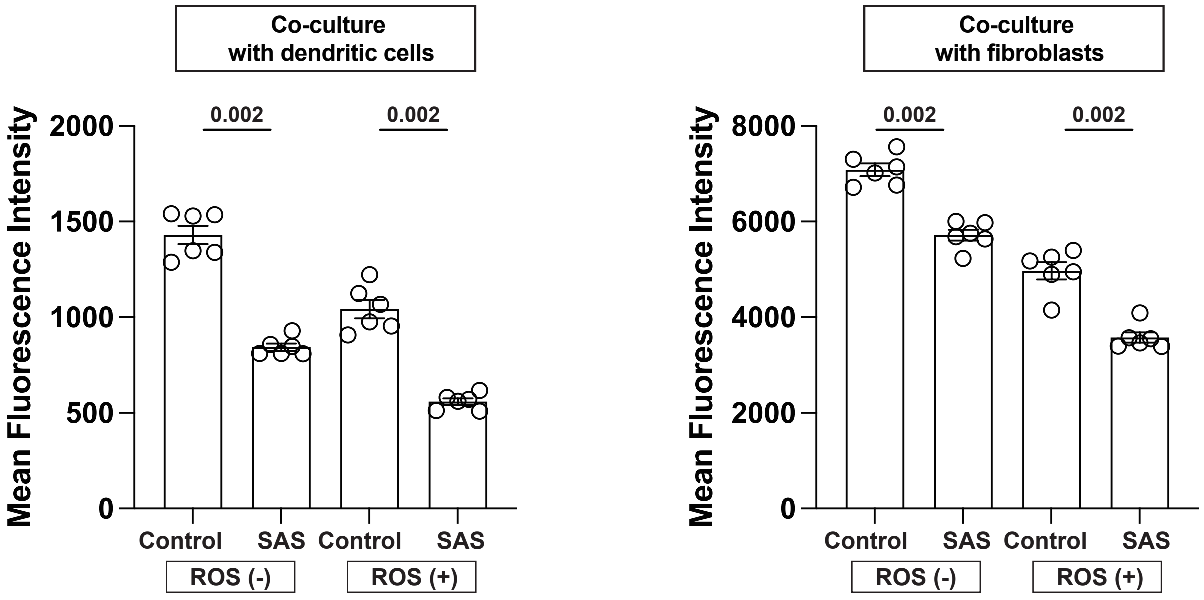

Supplemental Figure 11. Intracellular glutathione levels of T cells co-cultured with dendritic cells or fibroblasts pre-treated with sulfasalazine.

The quantitative MFI of ThiolTracker Violet in T cells co-cultured with dendritic cells or fibroblasts treated with sulfasalazine or vehicle (n = 6 in each group).

Values are means ± SE. Data were analyzed by Mann-Whitney U test.

SAS, sulfasalazine.

Supplemental Figure 12

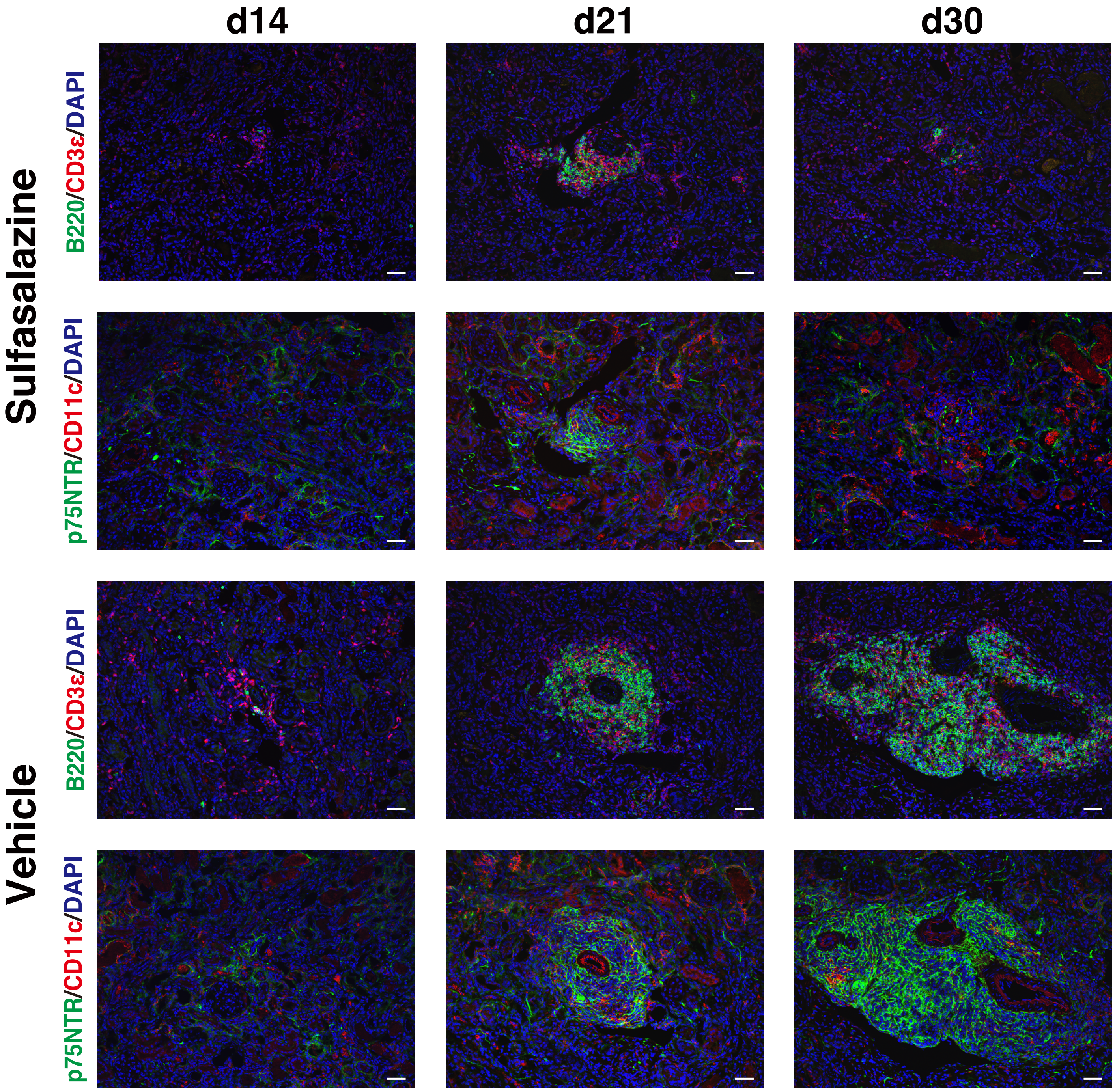

Supplemental Figure 12. Time course immunostaining of the kidneys treated with sulfasalazine or vehicle after IRI.

Immunofluorescence of B220 (green), CD3ε (red), and DAPI (blue) and p75NTR (green), CD11c (red), and DAPI (blue) in the kidneys at Day14, Day21, and Day30 after IRI treated with sulfasalazine or vehicle. Scale bars: 50 μm.

Supplemental Figure 13

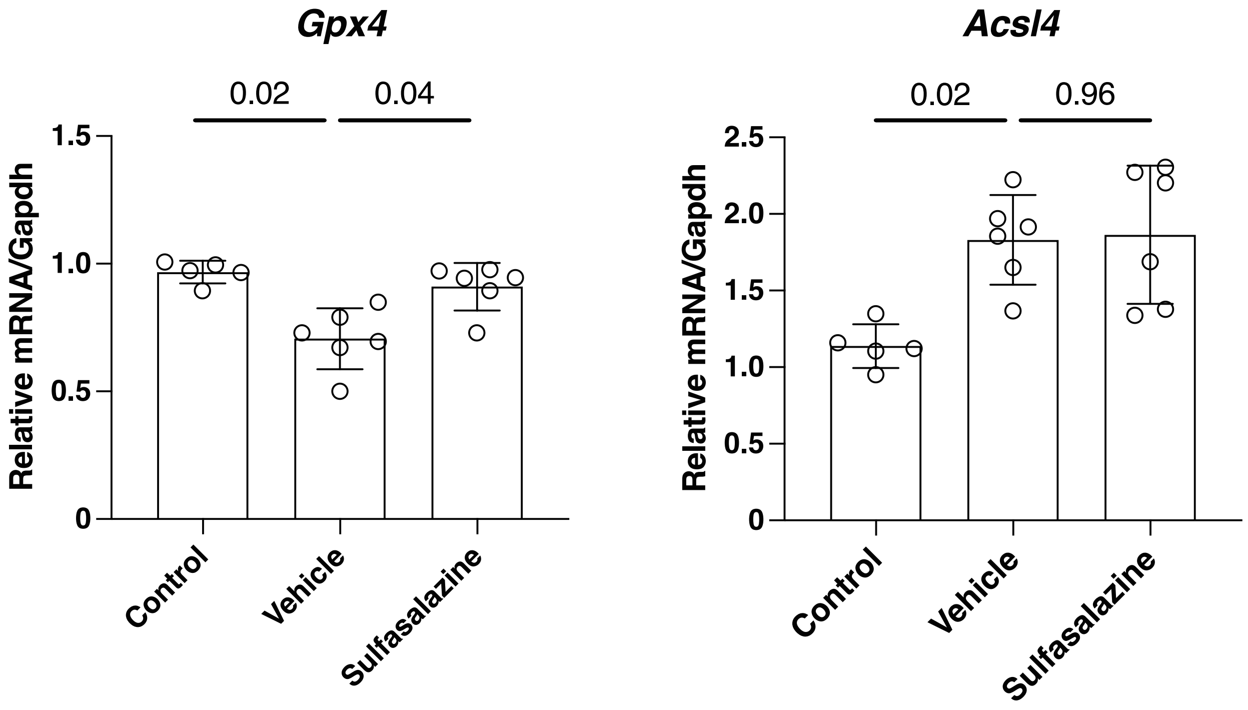

Supplemental Figure 13. Alterations of *Gpx4* and *Acsl4* expression following sulfasalazine treatment.

Gpx4 and *Acsl4* mRNA levels in the IRI kidneys (Control: kidneys 30 days after sham operation, n = 5; Vehicle: kidneys 30 days after IRI treated with vehicle from day14, n = 6; Sulfasalazine: the kidneys 30 days after IRI treated with sulfasalazine 400 mg/kg from day 14, n = 6). Values are means ± SE. Data were analyzed by Steel test with the vehicle-treated group as control.

Supplemental Figure 14

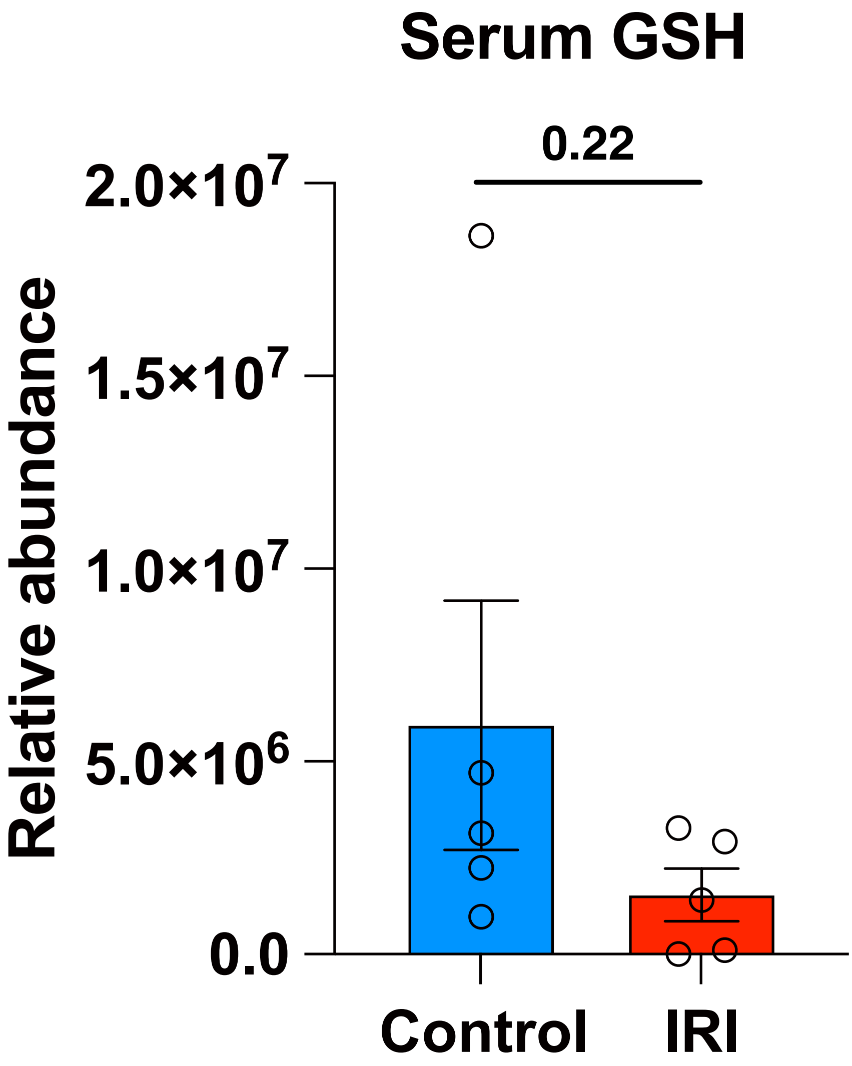

Supplemental Figure 14. Serum GSH concentrations do not increase in mice with tertiary lymphoid structures in the kidney.

Serum GSH levels of aged mice at day 45 after IRI and sham surgeries (n = 5 in each group).

GSH levels were measured by mass spectrometry. Values are means ± SE.

Data were analyzed by Mann-Whitney U test. GSH, Glutathione reduced form.

Supplemental Figure 15

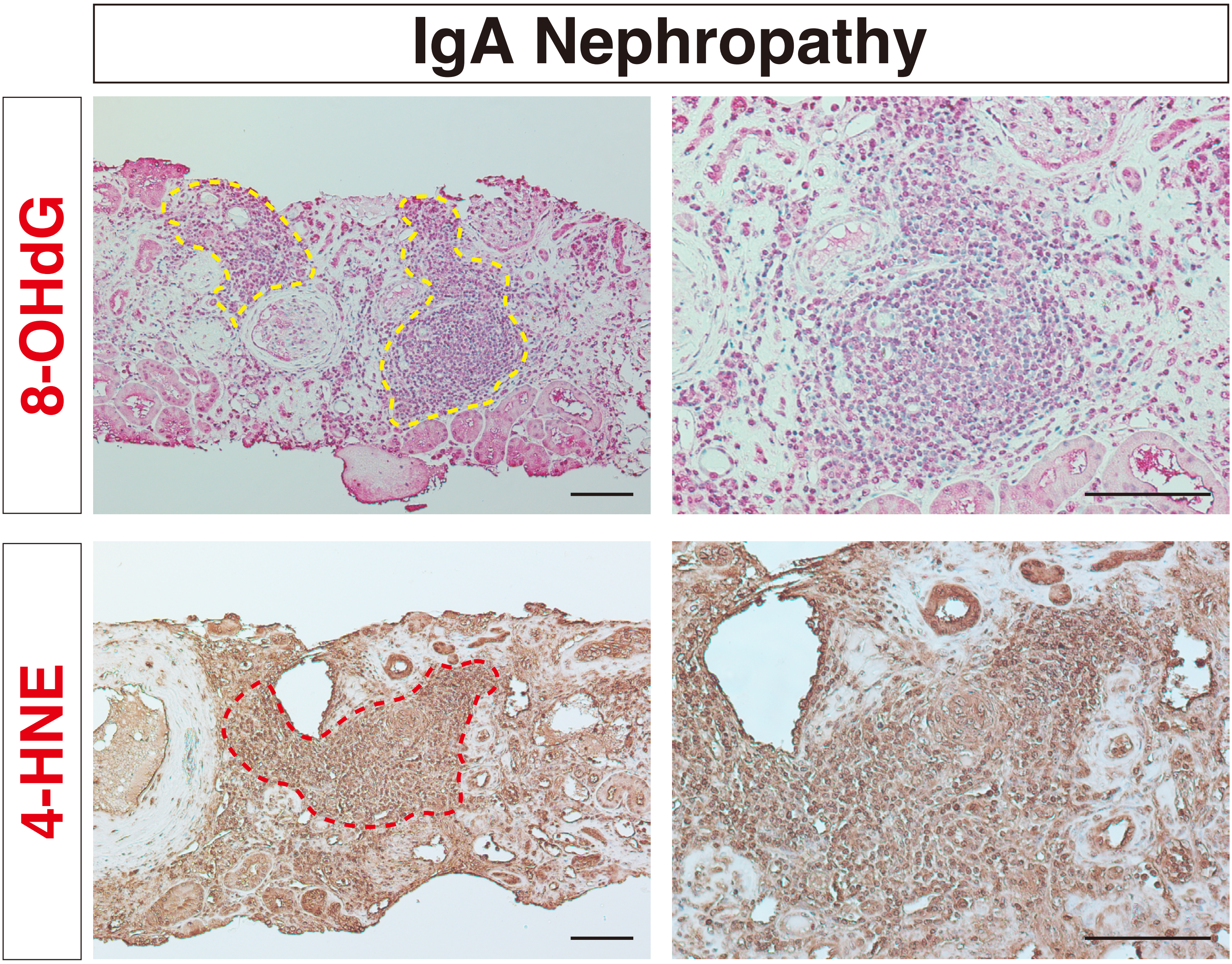

Supplemental Figure 15. 8-OHdG and 4-HNE accumulate within tertiary lymphoid structures in the kidney of IgA nephropathy patients.

Immunohistochemistry of 8-OHdG and 4-HNE in kidney biopsy samples of IgA nephropathy patients with TLSs. 8-OHdG and 4-HNE were detected by alkaline phosphatase (red) and DAB (brown) staining, respectively. Sections were counterstained with eosin. Tertiary lymphoid structures are encircled by dotted lines.

Scale bars: 50 μm.

Supplemental Figure 16

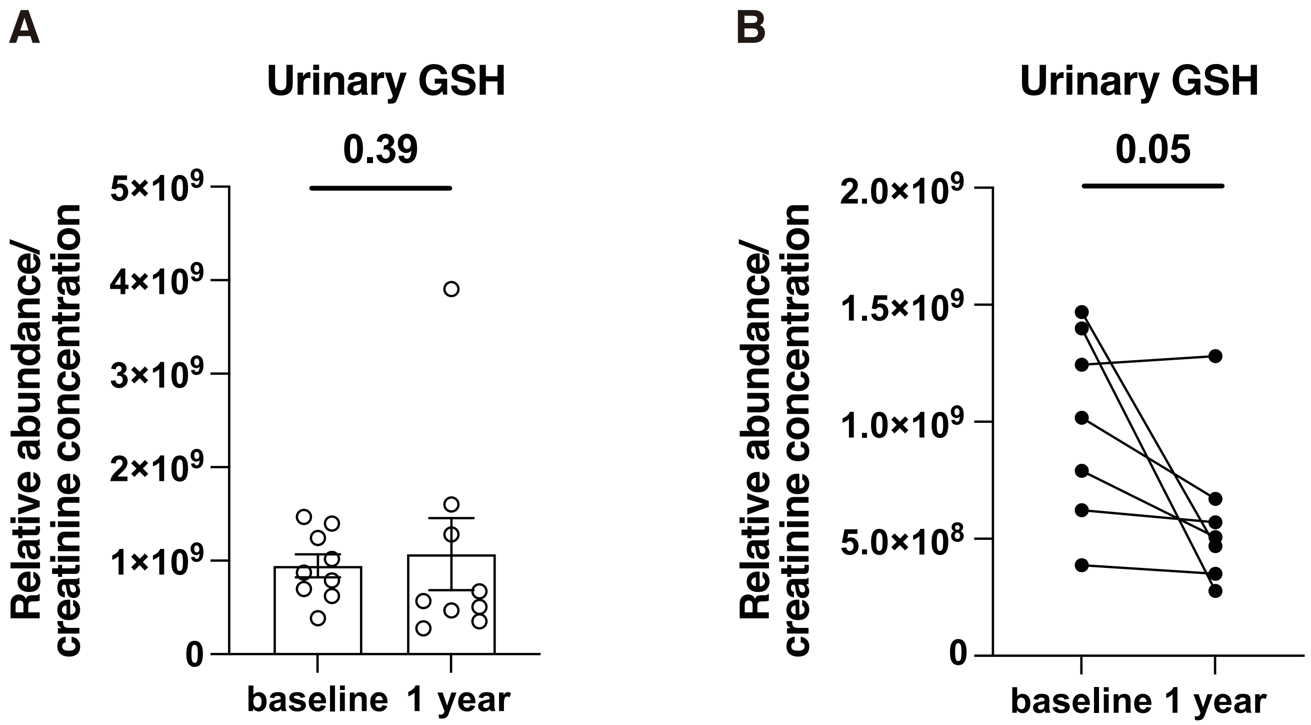

Supplemental Figure 16. Urinary GSH concentrations at baseline and one year after diagnosis in IgA nephropathy patients with tertiary lymphoid structures.

(A) GSH levels in urine samples from IgA nephropathy patients with tertiary lymphoid structures in the kidney. Nine patients were recruited from those with tertiary lymphoid structures shown in Figure 9, who underwent steroid treatment, with urine samples available for analysis at both diagnosis and one year later.

(B) Comparison of urinary glutathione levels at baseline and one year later among seven patients who exhibited more than 50% decrease in urinary protein levels following steroid treatment.

Values are presented as means ± SE. Data were analyzed by (A, B) Wilcoxon signed-rank sum test.

GSH, Glutathione reduced form.

Supplemental Figure 17

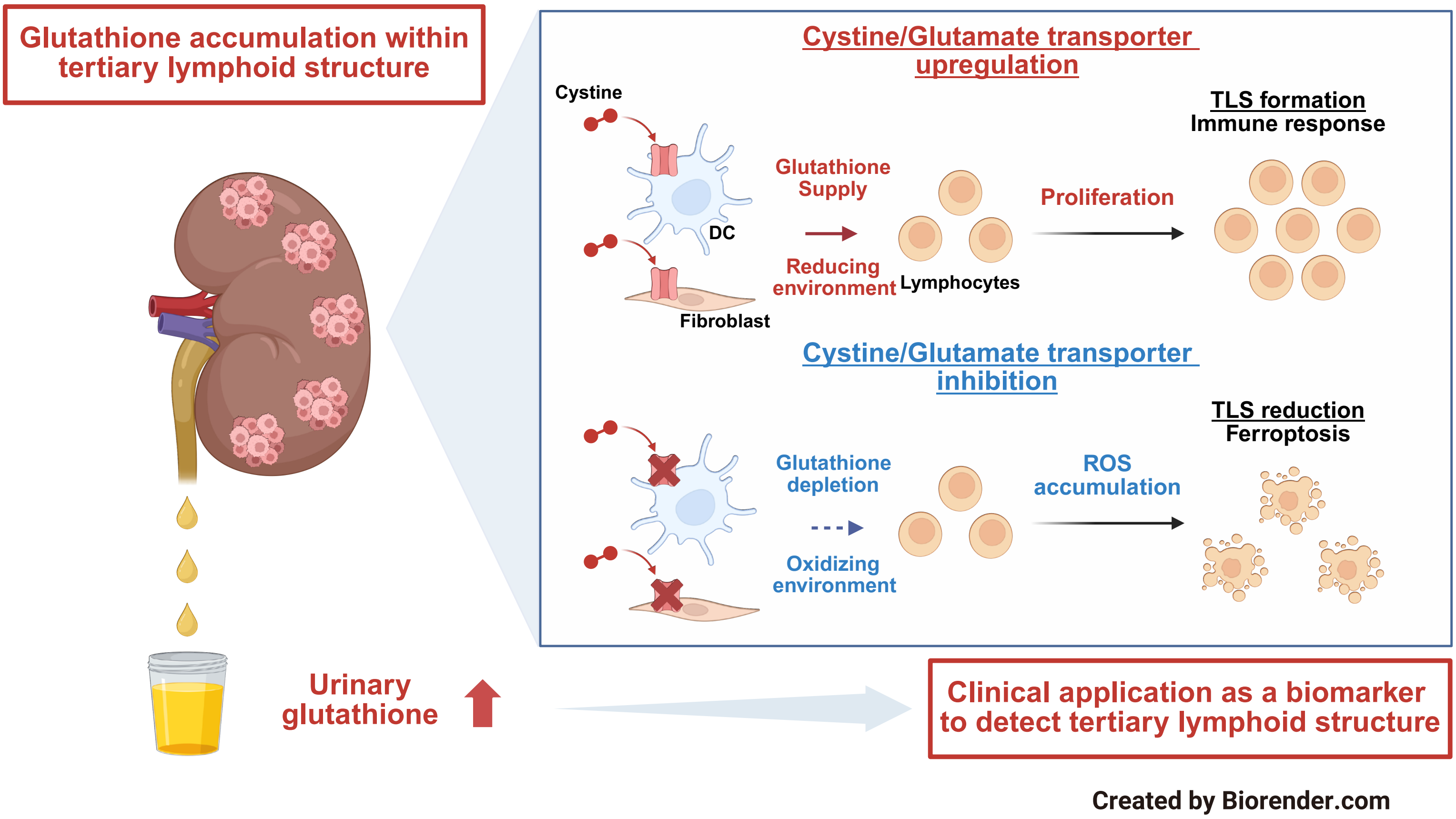

Supplemental Figure 17. A scheme showing metabolic microenvironment of TLS and clinical potential of urinary glutathione as a biomarker to detect TLS in the kidney.

Our study unveiled a unique metabolic microenvironment within tertiary lymphoid structures in the kidney. The distinctive metabolic signature of tertiary lymphoid structures is the accumulation of glutathione. The glutathione synthesis pathway plays a pivotal role in the formation of tertiary lymphoid structures, acting as a resilience mechanism to oxidative stress and ferroptosis. Urinary glutathione holds important clinical promise as a biomarker for identifying tertiary lymphoid structures in the kidney.
